# Gene Regulation by CcpA and Catabolite Repression Explored by RNA-Seq in *Streptococcus mutans*

**DOI:** 10.1371/journal.pone.0060465

**Published:** 2013-03-28

**Authors:** Lin Zeng, Sang Chul Choi, Charles G. Danko, Adam Siepel, Michael J. Stanhope, Robert A. Burne

**Affiliations:** 1 Department of Oral Biology, College of Dentistry, University of Florida, Gainesville, Florida, United States of America; 2 Department of Population Medicine and Diagnostic Sciences, College of Veterinary Medicine, Cornell University, Ithaca, New York, United States of America; 3 Department of Biological Statistics and Computational Biology, Cornell University, Ithaca, New York, United States of America; University of Kansas Medical Center, United States of America

## Abstract

A bacterial transcriptome of the primary etiological agent of human dental caries, *Streptococcus mutans*, is described here using deep RNA sequencing. Differential expression profiles of the transcriptome in the context of carbohydrate source, and of the presence or absence of the catabolite control protein CcpA, revealed good agreement with previously-published DNA microarrays. In addition, RNA-seq considerably expanded the repertoire of DNA sequences that showed statistically-significant changes in expression as a function of the presence of CcpA and growth carbohydrate. Novel mRNAs and small RNAs were identified, some of which were differentially expressed in conditions tested in this study, suggesting that the function of the *S. mutans* CcpA protein and the influence of carbohydrate sources has a more substantial impact on gene regulation than previously appreciated. Likewise, the data reveal that the mechanisms underlying prioritization of carbohydrate utilization are more diverse than what is currently understood. Collectively, this study demonstrates the validity of RNA-seq as a potentially more-powerful alternative to DNA microarrays in studying gene regulation in *S. mutans* because of the capacity of this approach to yield a more precise landscape of transcriptomic changes in response to specific mutations and growth conditions.

## Introduction

About one fifth of children between the ages of 2 and 19 were reported to have untreated dental caries in the United States (National Center for Health Statistics, 2010). It is generally accepted that the presence of acid-tolerant bacteria, a carbohydrate-rich diet and a susceptible host are all required for development of dental caries. In fact, carbohydrates introduced into the oral cavity provide the preferred energy sources for the majority of the most abundant members of the oral microbiome. This is especially true for those organisms that are regarded as significant contributors to the caries process, including aciduric streptococci, certain *Actinomyces* spp., and various lactobacilli, bifidobacteria and *Scardovia* spp. These organisms are particularly effective at converting host- and diet-derived carbohydrates into the organic acids that can directly effect demineralization of the tooth [1], [2], [3].

Cariogenic bacteria, including the primary etiological agent of human dental caries, *Streptococcus mutans*, are usually equipped with multiple pathways for the internalization and catabolism of carbohydrates [4]. The development of these repertoires of carbohydrate catabolic pathways likely reflects adaptation to the complex combination of carbohydrates that are secreted by the host in the glycoproteins and other glycoconjugates produced in saliva and gingival exudates, as well as to the variety of simple and complex carbohydrates that became more significant components of the human diet a few thousand years ago. Because of the complexities of the repertoire of carbohydrates to which oral biofilms are exposed and the intermittent feeding patterns of humans, it is reasonable to conclude that many of the most abundant members of the oral microbiota have evolved sophisticated pathways to rapidly and efficiently prioritize the assimilation, catabolism and storage of carbohydrates. Likewise, when the diet becomes enriched for carbohydrates, the microorganisms must adapt their utilization of substrates as the physico-chemical and microbial composition of the biofilms acquire the characteristics associated with enhanced cariogenic potential. These characteristics include enrichment for the aforementioned aciduric organisms, lower biofilm pH and other changes in microenvironments, e.g. reduced redox, which can alter bacterial gene expression and physiology [5], [6], [7], [8].

Carbon catabolite repression (CCR) allows bacteria to utilize carbon sources in a selective fashion, turning off non-essential catabolic functions while activating pathways required for utilization of preferred carbohydrates and other carbon sources [9], [10]. In a number of low-G+C Gram-positive pathogenic bacteria, CCR has also been shown to affect the expression of numerous virulence factors in response to the source and amount of carbohydrate [9]. CCR in these organisms is primarily exerted through the catabolite control protein A, CcpA. When preferred carbohydrate sources are present, as sensed through the accumulation of glycolytic intermediates, a protein kinase (HprK) is activated that can phosphorylate the general sugar:phosphotransferase system (PTS) protein HPr at serine 46 (HPr-Ser46-PO_4_). Hpr-Ser46-PO_4_ functions as a co-factor for the binding of CcpA to conserved catabolite response elements (CRE) found near the start sites of target genes to activate or repress gene expression, depending on the gene, the position of the CRE and other factors [10].

CcpA was shown to regulate carbohydrate metabolism and virulence expression in *S. mutans* in a transcriptomic study using a Microarray technique [11]. A Regprecise search (http://regprecise.lbl.gov) of the *S. mutans* UA159 genome yielded 99 genes in 48 operons with potential CREs detected in their promoter regions [12]. Although only a very small number of these genes have been confirmed to be regulated by CcpA in *S. mutans*, apparent homologues in related bacteria have been shown to be subject to CCR by CcpA. A comparison with the results of our previous Microarray analysis indicated that, using a two-fold cutoff, about half of the operons predicted by Regprecise were not among the genes found to be differentially expressed in a *ccpA* mutant [11]. Notably, in another study using Microarrays that included all intergenic regions (IGR) of *S. mutans* UA159, differential expression of certain IGRs was observed in cell sub-populations that responded to the bacterial quorum-sensing signal CSP (competence-stimulating peptide) [13]. Some of these IGRs may encode as-yet-uncharacterized regulatory RNAs or encode proteins that may help regulate the development of competence, biofilm formation and stress tolerance in *S. mutans*. Similarly, as-yet-undisclosed IGRs or small RNAs in the genome of *S. mutans* could play regulatory roles in carbohydrate metabolism.

Microarray analysis has proven to be reliable, rapid and comparatively economical method to analyze bacterial transcriptomes [14]. However, only genes or transcripts that are included in a predetermined set of probes can be detected in any given assay. Thus, the technique generally does not capture unannotated transcripts or genes, or is of limited use for strains with a different complement of non-core genes than the sequenced reference strain(s). Relatively recently, RNA deep sequencing (RNA-seq) has facilitated annotation- and probe-free detection of bacterial transcripts, with greater sensitivity and dynamic range in RNA expression levels than Microarrays [15], [16]. In addition, RNA-seq allows for the analysis of RNAs in non-coding regions, of small RNAs (sRNAs) and of antisense transcripts. However, as a relatively new tool in modern molecular microbiology, further validation is needed to ensure that confounding variables do not bias the results to a significant degree. Confounders include bias in steps that require reverse transcription and ligation and, particularly for bacterial samples, relatively low signal/noise ratios due to the presence of a large ribosomal RNA population. In order to test the applicability of RNA-seq for transcriptomic studies in *S. mutans,* we adopted RNA deep sequencing techniques to sequence enriched mRNAs from 13 *S. mutans* samples, focusing on mRNA and sRNA levels in response to different carbohydrates (glucose and galactose) in both the wild-type strain (UA159) and a *ccpA* mutant (TW1) [11]. As we have previously conducted Microarray analysis with the same set of strains grown under identical conditions, we could validate the RNA-seq technology while expanding our knowledge of the scope of RNAs that may play a role in regulation of carbohydrate metabolism, a central factor in *S. mutans* virulence.

## Materials and Methods

### Bacterial strains and growth conditions

*Streptococcus mutans* strains UA159 and TW1 [11] were maintained on BHI (Difco Laboratories, Detroit, MI) agar plates, and bacterial cultures used for extraction of RNA were prepared with Tryptone-vitamin [17] base medium supplemented with 0.5% of glucose or galactose (Sigma, St. Louis, MO). Four repeats, each in a volume of 15 mL, were included for the culture of strain UA159 growing in TV-galactose, while 3 repeats were used for each of the other 3 cultures. Bacterial cultures were incubated statically in the presence of 5% CO_2_ at 37°C until they reached mid-exponential phase (OD_600_≈0.5), harvested by centrifugation at 4°C for 10 min, treated with bacterial RNAprotect Bacteria Reagent (Qiagen, Germantown, MD), and immediately stored at −80°C.

### RNA isolation, mRNA enrichment and sequencing

Total RNA was extracted from bacterial cells using the RNeasy Mini kit (Qiagen) according to previously published protocols [18]. To remove 16S and 23S rRNAs, 10 µg of high-quality total RNA was processed using the MICROB*Express*™ Bacterial mRNA Enrichment Kit (Ambion of Life Technologies, Grand Island, NY), twice, before precipitating with ethanol and resuspending in 25 µL of nuclease-free water. The final quality of enriched mRNA samples was analyzed using an Agilent Bioanalyzer (Agilent Technologies, Santa Clara, CA). cDNA libraries were generated from the enriched mRNA samples using the TruSeq Illumina kit (Illumina, San Diego, CA), following instructions from the supplier. Deep sequencing was performed at the Cornell University Life Sciences Core Laboratories Center (Ithaca, NY).

### Short-read alignments

Approximately 20 million short-reads were obtained for each sample. Because the aligner BWA [19] allowed a few gaps for efficient alignment of millions of reads of approximately 100 bp, shorter reads consisting mostly of sequencing adapters would not be mapped. After removing adapter sequences from each short-read [20] and trimming of the 3′-ends by quality scores [21], the resulting sequences were mapped onto the reference genome of strain UA159 (GenBank accession no. AE014133) using the short-read aligner. Mapped short-read alignments were then converted into readable formats using SAMTOOLS [22].

### Transcript predictions

RNA transcripts were inferred by applying a hidden Markov model to site-wise expression levels [23]. A pileup command of BWA was used to convert the short-read alignments into pileup values, which were taken as the site-wise expression levels along the genome. Site-wise expression levels are a list of non-negative integers that represent numbers of short-reads mapped to a particular genomic position. For the transcript inference program, ParseRNAseq, the following options were used: “-c 10 -b 25 -force gp”, which binned expression levels into 25 parts and allowed 10 emission states for relative expression intensity. Genes that were annotated in close proximity were used as the predicted parts of a transcript, while no information regarding the precise transcription initiation- or stop-sites were pursued in this study. For similar reasons, the orientation of each transcript could not be verified solely based on RNA-seq data; instead we used the information of annotated genes to determine the strandedness of predicted transcripts.

### Prediction of small RNAs and targets

Although our RNA-seq protocol did not specifically enrich non-coding small RNAs in the cDNA preparation, small RNAs were retained in the RNA samples as only ribosomal RNAs were depleted using specific oligonucleotides. Consequently, it was difficult to discriminate cDNA originated from small non-coding RNAs from that of mRNAs, as expression of non-coding RNA is often masked by the expression of neighboring background mRNAs. Therefore, we utilized RNAz [24], a program that uses homologous sequences and RNA secondary structures to predict putative non-coding small RNAs. Because sequence alignment was a critical step for finding small RNAs via this approach, intergenic regions that also included up- and down-stream sequences were extracted, BLAST-searched against a database of bacterial genomes [25], and the resultant sequence alignments were further refined using a program named MUSCLE [26]. Subsequently, RNAz was applied to the alignments for scoring intergenic regions for putative small RNAs [24], [27]. Targets genes for each candidate small RNA were predicted using RNAplex [28] and RNAplfold [29], and the resultant genes were then used to perform functional category enrichment tests based on their scores by these two programs. In addition, we employed a method of Rho-independent terminator (RIT) identification to help identify candidate small RNAs [30], which were subsequently scored using RNAz, and a transcriptional signal-based method to identify intergenic sRNA transcription units (TUs) [31].

### Statistical analysis for differential expression

The R package DESeq [32] was used to determine differential gene expression on the basis of the negative binomial model [33]. Detailed steps for analyzing RNA-seq data for differentially expressed genes were utilized as described elsewhere [34]. Briefly, short-reads aligned to a particular annotated gene in the reference genome were counted, generating a table of read counts of all the open-reading frames. Statistical software R of the R package DEseq [35] was then employed to infer differentially expressed genes in various biological conditions. To normalize expression levels among different samples, total sequencing depths for each sample were estimated as the median of the ratios of the sample's counts to geometric mean across all samples, as detailed elsewhere [32], [36].

### Gene functional category associations

Three sets of gene categories were compiled for testing functional associations of differentially expressed genes. First, genes were each assigned to Gene Ontology (GO) categories by comparing to bacterial proteins from the Uniref90 database using the BLASTP program, and a GO classification was assigned if the match had an *E*-value of<1.0×10^−5^. A gene family was assigned a given classification if any of its genes was assigned that classification. Second, this classification was collated against functional classes of genes in the Oral Pathogen Sequence Databases available at http://oralgen.lanl.gov. Third, genes were mapped onto a set of metabolic pathways for *S. mutans* available at Kyoto Encyclopedia of Genes and Genomes (KEGG) [37]. Using the sets of gene categories, a variation of Fisher's exact test was conducted accounting for gene lengths to test enrichment of differentially expressed genes [38]. To determine the functional categories of predicted target genes of small RNAs, a Mann-Whitney *U* test was performed using the values of the target genes in association with a given category versus those for the other categories. Both tests were corrected for multiple testing hypotheses [39].

### UCSC genome browser tracks and data availability

Tracks were created for the recently-released Streptococcus Genome Browser (http://strep-genome.bscb.cornell.edu) that summarized the results of our *S. mutans* transcriptomic analysis with known genes, gene expression levels based on the short-read alignments, predicted putative transcripts, and predicted small non-coding RNAs. These results can be accessed by clade “*Streptococcus*”, genome “*S. mutans* UA159” and assembly “January 2006”. These tracks can be used to inspect loci of interest and to compare the results of different RNA-seq data sets. They can also be queried and intersected with other tracks using the UCSC Table Browser.

## Results and Discussion

### Clustering of RNA samples

**Figure 1:**
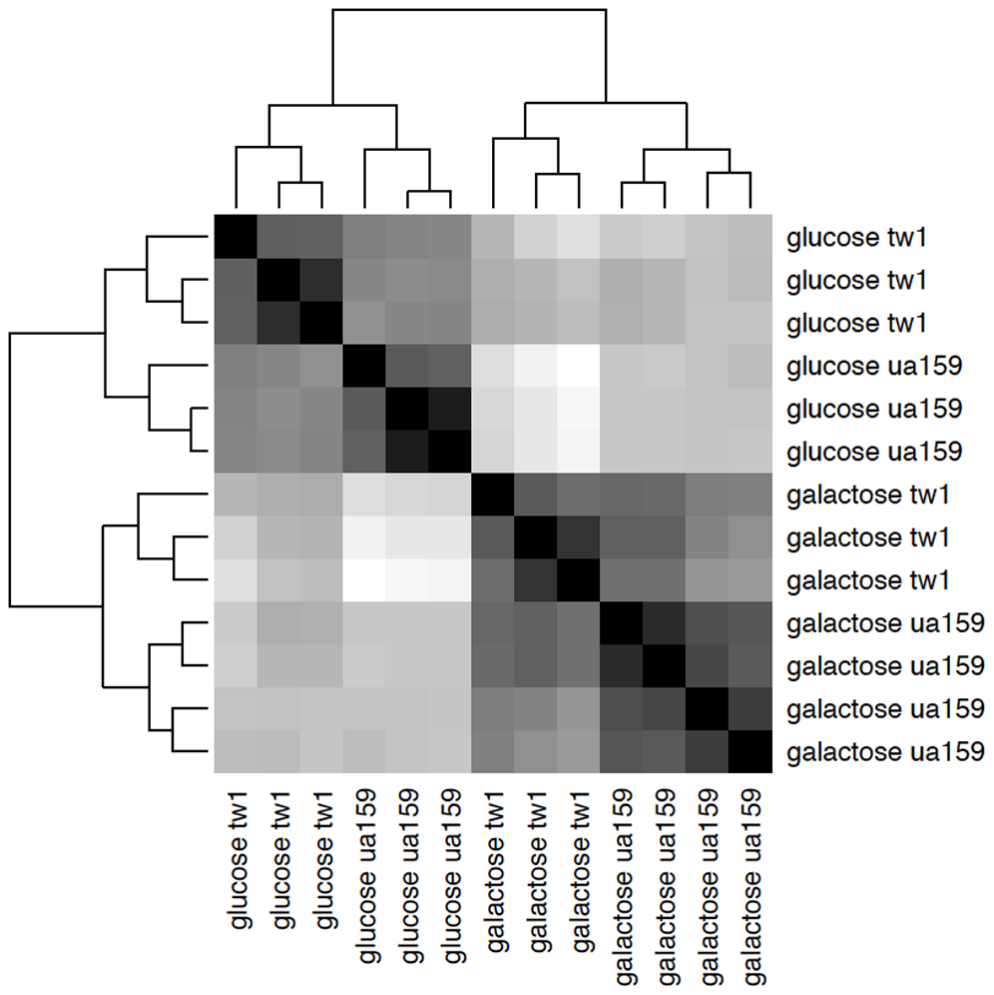
Clustering of 13 RNA-seq samples. Heatmap shows the Euclidean distances between the samples as calculated from the variance-stabilizing transformation of the count data.

Based on the results of read counts of all annotated genes, a total of 13 RNA-seq samples were clustered without supervision. As illustrated in Figure 1, the effect of carbohydrate source appeared to be significantly stronger than that of the loss of the *ccpA* gene. Nevertheless, all replicates of the same bacterial strain growing in the same carbohydrate conditions clearly clustered together, indicating that the transcriptomic shifts were the results of both sugar specificity and CcpA.

### Predicted transcripts

**Figure 2:**
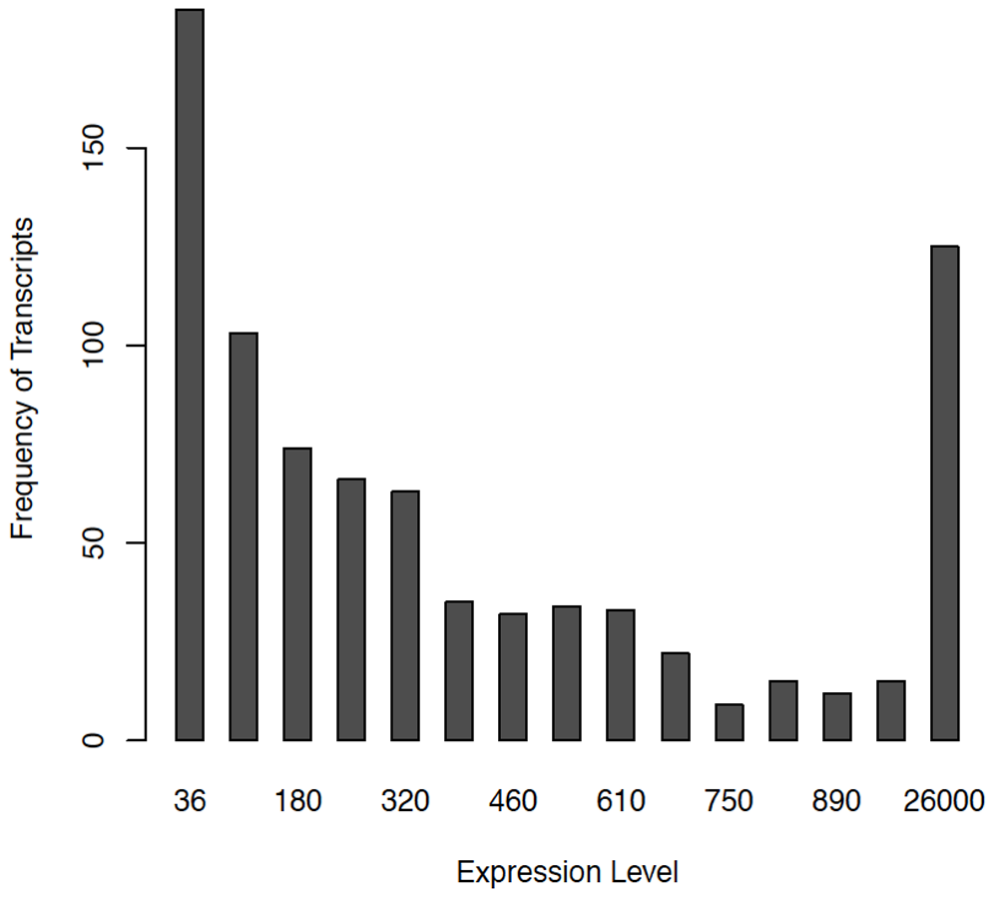
Distribution of expression levels of the predicted transcripts. The last bin sums from the expression levels 1000 to 50,000. The expression levels were measured as the average of reads mapped on predicted transcripts.

**Figure 3:**
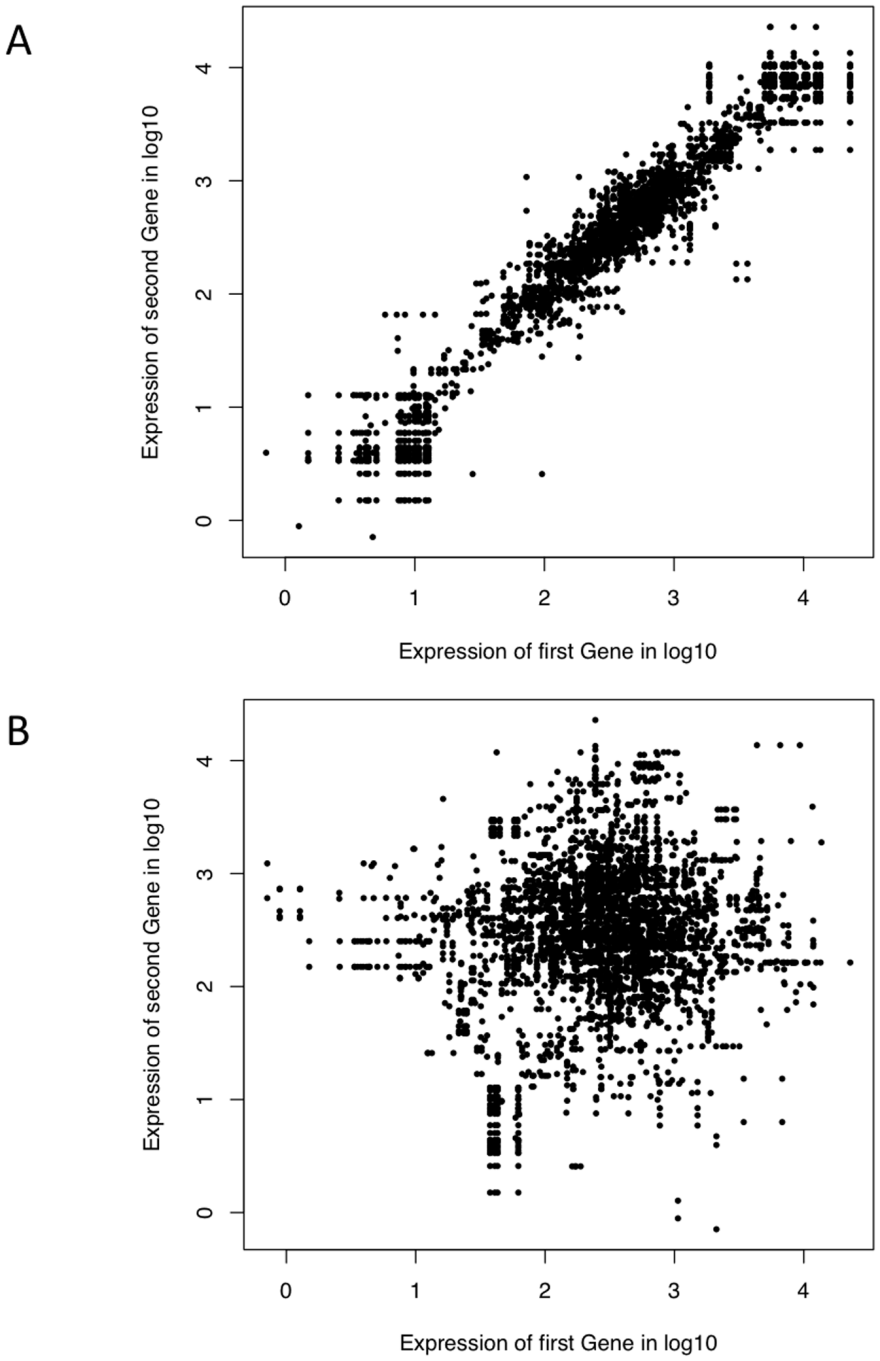
Scatter plot of the expression levels of pairs of adjacent genes. The expression levels of two genes located within the same transcript (A) or separate but adjacent transcripts (B) are plotted in log_10_ scale.

Using the RNA-seq data, a set of transcripts were obtained for each of the 13 samples. In order to summarize the 13 sets of predicted transcripts, all transcripts were scored based on their average site-wise expression levels in relation to transcripts in the rest of the set. More highly-ranked segments were placed first on the reference genome based on their scores, while lower-ranked segments were purged from the transcriptome map. In doing so, a final set of data were generated as non-overlapping transcripts. The distributions of expression levels of the predicted transcripts are shown in Figure 2. Designated as expressed were transcripts with average site-wise expression levels greater than 5 and with proportion of sites with zero site-wise expression of less than 10%. A gene was designated as expressed if it belonged to an expressed transcript. Of 823 predicted transcripts, 11 were found either expressed poorly or not expressed at all; and of the total 1960 genes, 1947 (99%) were designated as expressed (Figure S1). Of the 812 expressed transcripts, 84 contained no annotated genes or RNAs. As a measure of quality of the transcriptome map, the expression levels between pairs of adjacent genes were compared and the results indicated that the expression levels for two genes located within the same predicted transcript (Figure 3A) were far better correlated than those belonging to two adjacent transcripts (Figure 3B).

Figure S2 shows the length distribution of expressed transcripts containing annotated genes and unannotated regions. Unannotated regions could include potential non-coding RNAs and novel genes. Transcripts from unannotated regions appeared to be generally shorter than those with annotated genes. We used strandedness of known genes to determine that of a hosting transcript as a strand-specific RNA-seq technique was not employed here. Of the 812 expressed transcripts, 204 included annotated genes with conflicting strandedness. Among the 608 transcripts without conflicting strandedness, 271 contained a single gene while 337 were polycistronic. Figure S3 shows length distributions of 5′ and 3′ untranslated regions (UTR) of the final 608 transcripts.

### Prediction of small RNAs and targets

Three different methods, RNAz, Rho-independent terminator-based and transcriptional signal-based identifications were used to predict the presence of small non-coding RNAs, yielding 105, 69 and 135 regions of interest, respectively. After eliminating overlapping hits among these predictions, a pool of 243 genomic regions was generated (Table S1). Because we focused our search of small RNAs on intergenic regions, only 3 predicted small RNA regions overlapped known genes. As an example, for each of the 105 small RNAs generated using RNAz, target genes were predicted using RNAplex and RNAplfold methods and the functional categories of those that met our criteria are summarized in Table 1. After analyzing the RNA-seq data for the expression levels of each predicted small RNA, it was found that 114 of the 243 predicted sRNAs were actively expressed in our samples. Ultimately, five regions met all criteria for differential expression by UA159 cells grown in glucose versus galactose: PredSmallRNA-35, PredSmallRNA-71, PredSmallRNA-116, PredSmallRNA-117, and PredSmallRNA-204 (see Figure S4 for MFE structure drawing). In the other three sets of pair-wise comparisons, we also found other differentially expressed regions: PredSmallRNA-204, and PredSmallRNA-205 (UA159/TW1, glucose condition); PredSmallRNA-35 (glucose/galactose, TW1 background); PredSmallRNA-116 and PredSmallRNA-205 (UA159/TW1, galactose condition). Interestingly, by investigating the expression patterns of the neighboring transcripts, an intergenic region that hosts the predicted small RNAs PredSmallRNA-204 and PredSmallRNA-205 was identified as being regulated in a fashion independent of the surrounding genes.

**Table 1:** Gene Ontology enrichment for target genes of putative unannotated RNAs, as predicted using RNAz.

Small RNA	q^a^	Count	GO	Description
**PredSmallRNA-233**	0.043	165	GO:0016021	integral to membrane
**PredSmallRNA-227**	0.00021	46	GO:0055085	transmembrane transport
**PredSmallRNA-223**	0.03	46	GO:0055085	transmembrane transport
**PredSmallRNA-222**	0.036	49	GO:0003735	structural constituent of ribosome
	0.036	51	GO:0005840	ribosome
	0.041	49	GO:0030529	ribonucleoprotein complex
**PredSmallRNA-49**	0.0041	49	GO:0030529	ribonucleoprotein complex
	0.0041	51	GO:0005840	ribosome
	0.0041	30	GO:0019843	rRNA binding
	0.0041	49	GO:0003735	structural constituent of ribosome
**PredSmallRNA-45**	0.016	46	GO:0055085	transmembrane transport
**PredSmallRNA-192**	0.0074	17	GO:0043169	cation binding
**PredSmallRNA-19**	0.015	197	GO:0003677	DNA binding
**PredSmallRNA-4**	0.02	10	GO:0048037	cofactor binding
**PredSmallRNA-171**	0.039	165	GO:0016021	integral to membrane
**PredSmallRNA-126**	0.02	10	GO:0048037	cofactor binding
**PredSmallRNA-139**	0.05	10	GO:0048037	cofactor binding
**PredSmallRNA-1**	0.011	101	GO:0005886	plasma membrane
	0.011	165	GO:0016021	integral to membrane
**PredSmallRNA-14**	0.006	10	GO:0048037	cofactor binding
**PredSmallRNA-10**	0.044	13	GO:0004519	endonuclease activity
**PredSmallRNA-8**	0.0016	10	GO:0048037	cofactor binding
**PredSmallRNA-5**	0.030	14	GO:0015074	DNA integration
	0.047	66	GO:0016301	kinase activity
	0.047	67	GO:0016310	phosphorylation

a False discovery rate estimated by the Benjamini-Hochberg method. Only categories having at least ten genes and q≤0.05 are displayed.

### Differential expression of mRNA transcripts

Two independent factors of the biological processes being studied here included the effects of possession of CcpA and of growth in glucose versus galactose, resulting in four pair-wise comparisons. The cut-off for designating a gene as being differentially expressed was a change in transcript levels of at least 2-fold and an adjusted *P*-value of less than 0.001. Of note, similar cut-off conditions were used in statistical analysis of our previously published Microarray assays [11]. Using a different threshold, namely 1.5-fold change in mRNA levels and an adjusted *P*-value of less than 0.05, a more extensive list of differentially expressed genes was identified (Table S8).

#### Comparison between wild-type and *ccpA* mutant strains in glucose-grown cells

**Figure 4:**
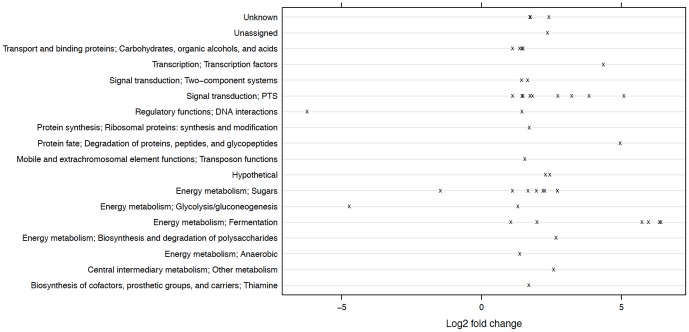
Distribution of functional classes of differentially expressed genes between UA159 and TW1 growing on glucose. The *x*-axis represents the log_2_ values of the fold of change in expression (TW1/UA159).

Table 2 lists differentially expressed genes in the comparison of wild-type UA159 and the *ccpA* mutant strain TW1 grown with glucose. Of 1960 genes, 45 genes showed at least a two-fold increase in expression in the mutant relative to the wild-type strain. At the same time, three genes showed at least a two-fold reduction in expression associated with the loss of CcpA. Among these differentially-expressed genes, 18 are involved in energy metabolism, including glycolysis, fermentation and sugar utilization; nine encode Enzyme II components of the PTS and are required for transporting glucose, mannose, sucrose or cellobiose; and four are classified as regulatory or two-component system genes (Figure 4 and S5; also see Tables S2 and S3 for gene ontology and KEGG terms that were enriched with differentially expressed genes in this comparison). We also found five differentially expressed genes annotated as hypothetical. We confirmed that the most up-regulated genes in TW1 encoded the components of the pyruvate dehydrogenase (PDH) enzyme complex (SMU.1421c∼1424c), as reported previously [11]. Down-regulated genes included a cytoplasmic α-amylase (SMU.1590) that is located downstream of *ccpA* [40], and a fructosyltransferase gene *ftf* (SMU.2028) encoding the enzyme involved in converting sucrose to a fructan homopolymer [41]. These results appeared to be generally consistent with those of the previous Microarray analysis [11]. To better contrast the RNA-seq results with that of Microarrays assays, genes that have been identified in corresponding microarrays are highlighted in Tables 2, 3, 4 and 5.

**Table 2:** List of genes differentially expressed in UA159 and TW1 when growing in TV-glucose.

Gene ID	log_2_(TW1/UA159)	*P*-value	Gene description
**SMU.1423c**	6.4	3E-148	putative pyruvate dehydrogenase, E1 component α-subunit
**SMU.1424c**	6.4	3E-151	putative dihydrolipoamide dehydrogenase
**SMU.1422c**	6.0	8E-135	putative pyruvate dehydrogenase, E1 component β-subunit
**SMU.1421c**	5.7	3E-102	branched-chain α-keto acid dehydrogenase subunit E2
SMU.1425c	5.0	2E-27	putative Clp proteinase, ATP-binding subunit ClpB
SMU.1600c	5.1	2E-10	cellobiose phosphotransferase system IIB component
SMU.1599c	4.3	7E-47	putative transcriptional regulator; possible antiterminators
SMU.1598c	3.8	2E-25	cellobiose phosphotransferase system IIA component
SMU.1596c	3.2	1E-39	cellobiose phosphotransferase system IIC component
SMU.1539c	2.7	1E-16	glycogen branching enzyme
SMU.1538c	2.7	3E-25	glucose-1-phosphate adenylyltransferase
**SMU.1537c**	2.3	3E-28	putative glycogen biosynthesis protein GlgD
SMU.1536c	2.2	2E-27	glycogen synthase
SMU.1535c	2.0	2E-22	glycogen phosphorylase
**SMU.2037c**	2.7	7E-34	putative trehalose-6-phosphate hydrolase TreA
**SMU.2038c**	2.7	8E-35	putative PTS system, trehalose-specific IIABC component
SMU.2127	2.6	2E-36	putative succinate semialdehyde dehydrogenase
**SMU.179**	2.4	1E-30	hypothetical protein
SMU.180	2.4	5E-31	putative oxidoreductase; fumarate reductase
**SMU.252**	2.4	2E-31	hypothetical protein
SMU.1862	2.3	6E-20	hypothetical protein
SMU.148	2.0	5E-12	bifunctional acetaldehyde-CoA/alcohol dehydrogenase
SMU.149	1.5	7E-11	putative transposase
SMU.1077	1.7	1E-16	putative phosphoglucomutase
SMU.1088	1.7	1E-16	putative thiamine biosynthesis lipoprotein
**SMU.1089**	1.7	3E-17	hypothetical protein
SMU.1090	1.7	7E-18	hypothetical protein
SMU.1841c	1.7	3E-17	putative PTS system, sucrose-specific IIABC component
SMU.1843	1.1	2E-06	sucrose-6-phosphate hydrolase
SMU.500	1.7	9E-17	putative ribosome-associated protein
SMU.576c	1.4	8E-11	putative response regulator LytR
**SMU.577c**	1.6	2E-15	putative histidine kinase LytS
SMU.1877	1.5	1E-07	putative PTS system, mannose-specific component IIAB
SMU.1878	1.8	1E-04	putative PTS system, mannose-specific component IIC
SMU.1879	1.4	5E-07	putative PTS system, mannose-specific component IID
SMU.1116c	1.5	4E-02	hypothetical protein
SMU.402c	1.4	2E-05	pyruvate formate-lyase
SMU.980	1.4	1E-09	putative PTS system, β-glucoside-specific EII component
SMU.981	1.1	2E-02	putative BglB fragment
SMU.982	1.2	2E-02	putative BglB fragment
**SMU.870**	1.4	5E-07	putative transcriptional regulator of sugar metabolism
SMU.871	1.3	2E-09	putative fructose-1-phosphate kinase
SMU.872	1.1	3E-07	putative PTS system, fructose-specific enzyme IIABC component
SMU.1004	1.2	1E-02	glucosyltransferase-I
SMU.1043c	1.0	2E-06	phosphotransacetylase
**SMU.2028c**	-1.5	9E-12	levansucrase precursor; β-D-fructosyltransferase
**SMU.1590c**	-4.7	6E-19	cytoplasmic α-amylase
**SMU.1591c**	-6.2	4E-29	catabolite control protein A, CcpA

**Bold font** indicates the gene has been identified by respective Microarray assay [11].

**Table 3:** List of genes differentially expressed in UA159 cells growing in TV-glucose (glc) and TV-galactose (gal).

Gene ID	log_2_(gal/glc)	*P*-value		Gene description				
**SMU.1498c**	3.5	2E-32		lactose repressor				
**SMU.1496c**	9.4	0E+00		galactose-6-phosphate isomerase subunit LacA				
**SMU.1495c**	9.2	8E-182		galactose-6-phosphate isomerase subunit LacB				
**SMU.1494c**	9.2	0E+00		tagatose-6-phosphate kinase				
**SMU.1493c**	9.1	0E+00		tagatose 1,6-diphosphate aldolase				
**SMU.1492c**	9	2E-122		PTS system, lactose-specific enzyme IIA EIIA				
**SMU.1491c**	8.3	0E+00		PTS system, lactose-specific enzyme IIBC EIIBC				
**SMU.1490c**	8.6	0E+00		6-phospho-β-galactosidase				
**SMU.1489c**	7.9	1E-295		LacX				
SMU.1488c	7.9	5E-249		hypothetical protein				
**SMU.1487**	1.3	2E-10		hypothetical protein				
SMU.1486c	1.1	4E-07		hypothetical protein				
**SMU.870**	1.1	1E-05		putative transcriptional regulator of sugar metabolism				
SMU.871	1.1	1E-09		putative fructose-1-phosphate kinase				
SMU.872	1.2	1E-12		putative PTS system, fructose-specific enzyme IIABC				
**SMU.882**	1.1	1E-09		multiple sugar-binding ABC transporter, MsmK				
SMU.885c	1.7	4E-20		galactose operon repressor GalR				
**SMU.886**	5.5	9E-135		galactokinase				
**SMU.887**	4.8	5E-150		galactose-1-phosphate uridylyltransferase				
**SMU.888**	3.6	1E-94		UDP-galactose 4-epimerase, GalE				
**SMU.889**	2.2	2E-40		putative penicillin-binding protein, class C; fmt-like protein				
SMU.1425c	3.9	3E-97		putative Clp proteinase, ATP-binding subunit ClpB				
**SMU.1424c**	3.6	5E-14		putative dihydrolipoamide dehydrogenase				
SMU.1423c	3.7	4E-12		putative pyruvate dehydrogenase, E1 component α-subunit				
SMU.1422c	3.5	1E-11		putative pyruvate dehydrogenase, E1 component β-subunit				
SMU.1421c	3.4	1E-12		branched-chain α-keto acid dehydrogenase subunit E2				
SMU.1600c	3.7	2E-16		cellobiose phosphotransferase system IIB component				
SMU.1599c	3.4	1E-39		putative transcriptional regulator; possible antiterminators				
SMU.1598c	3.2	1E-12		cellobiose phosphotransferase system IIA component				
SMU.1596c	2.7	3E-31		cellobiose phosphotransferase system IIC component				
**SMU.1539c**	2.9	6E-31		glycogen branching enzyme				
SMU.1538c	3.2	1E-77		glucose-1-phosphate adenylyltransferase				
**SMU.1537c**	3.1	2E-45		putative glycogen biosynthesis protein GlgD				
**SMU.1536c**	2.9	4E-67		glycogen synthase				
**SMU.1535c**	2.7	7E-60		glycogen phosphorylase				
SMU.2127	2.8	4E-62		putative succinate semialdehyde dehydrogenase				
SMU.1004	2.6	7E-14		glucosyltransferase-I				
SMU.1005	1.0	4E-10			glucosyltransferase-Si			
**SMU.2037c**	2.6	2E-41		putative trehalose-6-phosphate hydrolase TreA				
**SMU.2038c**	2.5	3E-38		putative PTS system, trehalose-specific IIABC component				
**SMU.180**	2.4	5E-49		putative oxidoreductase; fumarate reductase				
**SMU.179**	2.3	2E-39		hypothetical protein				
**SMU.252**	2.2	9E-24		hypothetical protein				
SMU.1862	2.1	5E-19		hypothetical protein				
SMU.112c	1.0	7E-05			putative transcriptional regulator			
SMU.113	2	9E-12		putative fructose-1-phosphate kinase				
SMU.114	1.8	4E-11		putative PTS system, fructose-specific IIBC component				
SMU.115	1.9	6E-09			putative PTS system, fructose-specific IIA component			
SMU.116	2	1E-14		tagatose 1,6-diphosphate aldolase				
SMU.1187c	2	6E-32		glucosamine–fructose-6-phosphate aminotransferase				
SMU.1185c	1.6	1E-15		PTS system, mannitol-specific enzyme IIBC component				
SMU.1396c	1.8	3E-16		glucan-binding protein C, GbpC				
SMU.1877	1.7	3E-23		putative PTS system, mannose-specific component IIAB				
SMU.1878	1.8	3E-07			putative PTS system, mannose-specific IIC component			
SMU.1879	1.6	9E-18		putative PTS system, mannose-specific component IID				
SMU.1116c	1.6	3E-13		hypothetical protein				
SMU.1117c	2.0	2E-07			NADH oxidase (H_2_O-forming)			
**SMU.402c**	1.9	1E-05			pyruvate formate-lyase			
SMU.270	1.2	2E-05			PTS system ascorbate-specific transporter subunit IIC			
SMU.1217c	1.1	8E-09			putative ABC transporter, amino acid binding protein			
SMU.980	1.5	3E-14		putative PTS system, β-glucoside-specific EII component				
SMU.1841c	1.4	4E-14		putative PTS system, sucrose-specific IIABC component				
SMU.1843	1.2	2E-12		sucrose-6-phosphate hydrolase				
SMU.1844	1.0	5E-09			sucrose operon repressor			
SMU.1088	1.1	9E-12		putative thiamine biosynthesis lipoprotein				
SMU.1090	1.1	2E-11		hypothetical protein				
SMU.1410	1.1	2E-06			putative reductase			
SMU.576c	1.0	2E-07			putative response regulator LytT			
SMU.577c	1.0	2E-08			putative histidine kinase LytS			
SMU.148	2	2E-36		bifunctional acetaldehyde-CoA/alcohol dehydrogenase				
SMU.149	1.1	2E-06			putative transposase			
SMU.915c	−1.1	4E-04			7-cyano-7-deazaguanine reductase			
SMU.916c	−1.1	7E-04			hypothetical protein			
SMU.917c	−1.2	1E-04			putative 6-pyruvoyl tetrahydropterin synthase			
SMU.954	−1.1	4E-07			pyridoxamine kinase			
SMU.1945	−1.3	2E-13		hypothetical protein				
SMU.1946	−1.1	2E-09			hypothetical protein			
SMU.1595c	−1.3	8E-09			putative carbonic anhydrase precursor			
SMU.423	−1.5	3E-09			possible bacteriocin NlmD			
**SMU.1175**	−1.6	1E-12		putative sodium/amino acid (alanine) symporter				
SMU.150	−1.9	1E-14		non-lantibiotic mutacin IV A, NlmA				
SMU.151	−1.8	6E-13		non-lantibiotic mutacin IV B, NlmB				
SMU.152	−2	1E-17		hypothetical protein				
SMU.153	−2.1	5E-37		hypothetical protein				
SMU.1898	−1.2	6E-10	putative ABC transporter, ATP-binding and permease protein					
SMU.1899	−1.3	4E-04	putative ABC transporter, ATP-binding and permease protein					
SMU.1902c	−1.8	9E-21		hypothetical protein				
SMU.1903c	−2.2	4E-17		hypothetical protein				
SMU.1904c	−2.2	4E-45		hypothetical protein				
SMU.1905c	−2.1	2E-17		putative bacteriocin secretion protein				
SMU.1906c	−2.1	2E-11		bacteriocin-related protein				
SMU.1907	−2.3	5E-28		hypothetical protein				
SMU.1908c	−2.2	5E-11		hypothetical protein				
SMU.1909c	−2.1	3E-32		hypothetical protein				
SMU.1910c	−2	4E-12		hypothetical protein				
SMU.1912c	−2	4E-14		hypothetical protein				
SMU.1913c	−2.1	1E-16		putative immunity protein, BLpL-like				
SMU.1914c	−2.0	2E-06	hypothetical protein					
**SMU.1700c**	−2.0	2E-04	LrgB family protein					
SMU.1701c	−2.0	2E-06	hypothetical protein					
**SMU.602**	−2.9	2E-06	putative sodium-dependent transporter					

**Bold font** indicates the gene has been identified by respective Microarray assay [11].

**Table 4:** List of genes differentially expressed in TW1 cells growing in TV-glucose (glc) and TV-galactose (gal).

Gene ID	log_2_(gal/glc)	*P*-value	Gene Description
**SMU.1498c**	3.3	9E-13	lactose repressor
**SMU.1495c**	9.4	2E-275	galactose-6-phosphate isomerase subunit LacB
**SMU.1496c**	9.2	3E-172	galactose-6-phosphate isomerase subunit LacA
**SMU.1494c**	9.2	4E-270	tagatose-6-phosphate kinase
**SMU.1493c**	9.1	1E-95	tagatose 1,6-diphosphate aldolase
**SMU.1492c**	9.1	6E-80	PTS system, lactose-specific enzyme IIA
**SMU.1491c**	8.2	5E-237	PTS system, lactose-specific enzyme IIBC
**SMU.1490c**	8.6	5E-250	6-phospho-β-galactosidase
**SMU.1489c**	7.8	1E-216	LacX
**SMU.1488c**	7.7	6E-203	hypothetical protein
**SMU.883**	1.3	8E-10	dextran glucosidase DexB
**SMU.882**	1.2	9E-08	multiple sugar-binding ABC transporter, MsmK
**SMU.881**	1.2	1E-07	sucrose phosphorylase, GtfA
**SMU.880**	1.1	2E-06	multiple sugar-binding ABC transporter, MsmG
**SMU.878**	1.1	2E-06	multiple sugar-binding ABC transporter, MsmE
SMU.877	1.0	5E-06	α-galactosidase
**SMU.1187c**	2.3	3E-31	glucosamine–fructose-6-phosphate aminotransferase
**SMU.1185c**	2.1	4E-18	PTS system, mannitol-specific enzyme IIBC
SMU.113	2.2	1E-09	putative fructose-1-phosphate kinase
SMU.114	1.8	2E-08	putative PTS system, fructose-specific IIBC
SMU.115	1.8	2E-04	putative PTS system, fructose-specific IIA
SMU.116	1.8	4E-05	tagatose 1,6-diphosphate aldolase
SMU.1004	1.7	4E-05	glucosyltransferase-I
**SMU.1896c**	1.6	8E-09	hypothetical protein
**SMU.1895c**	1.6	2E-06	hypothetical protein
**SMU.1396c**	1.4	3E-04	glucan-binding protein C, GbpC
SMU.1486c	1.3	3E-07	hypothetical protein
**SMU.1958c**	1.1	2E-05	putative PTS system, mannose-specific IIC
**SMU.1538c**	1.0	3E-06	glucose-1-phosphate adenylyltransferase
SMU.916c	−1.1	3E-04	hypothetical protein
**SMU.1898**	−1.1	3E-04	putative ABC transporter
SMU.919c	−1.2	1E-05	putative ATPase, confers aluminum resistance
SMU.152	−1.5	9E-05	hypothetical protein
SMU.1903c	−1.9	7E-05	hypothetical protein
**SMU.1905c**	−1.5	2E-04	putative bacteriocin secretion protein
SMU.1910c	−1.6	5E-04	hypothetical protein

**Bold font** indicates the gene has been identified by respective Microarray assay [11].

**Table 5:** List of genes differentially expressed in UA159 and TW1 when growing in TV-galactose.

Gene ID	log_2_(TW1/UA159)	*P*-value	Gene Description
**SMU.1425c**	1.0	2E-10	putative Clp proteinase, ATP-binding subunit ClpB
**SMU.1424c**	3.6	2E-15	putative dihydrolipoamide dehydrogenase
SMU.1423c	3.5	5E-16	putative pyruvate dehydrogenase, E1 component α-subunit
**SMU.1422c**	3.3	7E-23	putative pyruvate dehydrogenase E1 component β -subunit
SMU.1421c	3.1	2E-20	branched-chain α-keto acid dehydrogenase E2
**SMU.602**	3.0	2E-23	putative sodium-dependent transporter
**SMU.600c**	2.0	5E-23	hypothetical protein
**SMU.1700c**	2.6	5E-34	LrgB family protein
**SMU.1175**	2.5	2E-30	putative sodium/amino acid (alanine) symporter
**SMU.500**	1.9	7E-17	putative ribosome-associated protein
**SMU.1658c**	1.5	4E-04	putative ammonium transporter, NrgA protein
SMU.913	1.1	3E-10	glutamate dehydrogenase
**SMU.2042c**	1.0	3E-08	dextranase precursor
SMU.609	1.0	1E-05	putative 40K cell wall protein precursor
**SMU.2028c**	−2.1	3E-07	levansucrase precursor; β-D-fructosyltransferase
**SMU.1590c**	−5.5	5E-132	cytoplasmic α-amylase
**SMU.1591c**	−6.8	1E-237	catabolite control protein A, CcpA

**Bold font** indicates the gene has been identified by respective Microarray assay [11].

In agreement with the *in silico* prediction provided by the Regprecise website, results from the RNA-seq study, but not that of Microarray study, identified the following as differentially expressed: genes encoding enzymes for pyruvate kinase (SMU.1190c; decreased by two-fold in TW1) and pyruvate-formate lyase (SMU.402c; increased by twofold in TW1), a putative thiamine biosynthesis lipoprotein (SMU.1088), SMU.1125c of a putative ribonucleoside-metabolism operon, a ribosome associated protein (SMU.500), the sucrose-6-phosphate hydrolase (SMU.1843) and sucrose-PTS EII (SMU.1841c) [42], the major glucose-PTS EII^Man^ (SMU.1877∼1879) [43], the cellobiose-PTS operon (SMU.1596c∼1600c) [44] and another β-glucoside-PTS EII (SMU.980) [45]. On the other hand, genes identified only in the Microarray study that matched the Regprecise predictions included: glucose-6-phosphate isomerase (SMU.307), a putative ribulose-monophosphate-PTS EIIC component (SMU.270) and a hypothetical protein (SMU.799c). There were six potential CcpA-regulated operons overlapping these two methods.

There also appeared to be evidence suggesting improved consistency using RNA-seq technology. For example, a proven CCR-sensitive operon, the glycogen biosynthesis *glg* cluster that spans SMU.1535c∼1539c [11], [46], was shown here by RNA-seq to be uniformly derepressed in strain TW1 growing on glucose, whereas Microarray analysis of the same strains grown in the same condition indicated that the expression of only one of the genes, SMU.1537c, was altered in a statistically significant way. The same pattern was true for an inducible fructose-PTS operon (SMU.870∼872). Furthermore, RNA-seq analysis, at a two-fold cutoff, showed fewer genes (3 out of a total of 48) with decreased expression in TW1 as compared to UA159, whereas Microarray analysis showed 110 genes with decreased expression and 61 genes with increased expression in TW1 at the same cutoff, when both were growing in TV-glucose. A similar pattern appeared when the analysis was repeated at a lower (1.5-fold) threshold (Table S8). Considering the collective evidence regarding CCR in Gram-positive bacteria, it is conceivable that CcpA exerts its function predominantly through negative regulation [10].

#### Comparison between glucose- and galactose-grown UA159 cells

**Figure 5:**
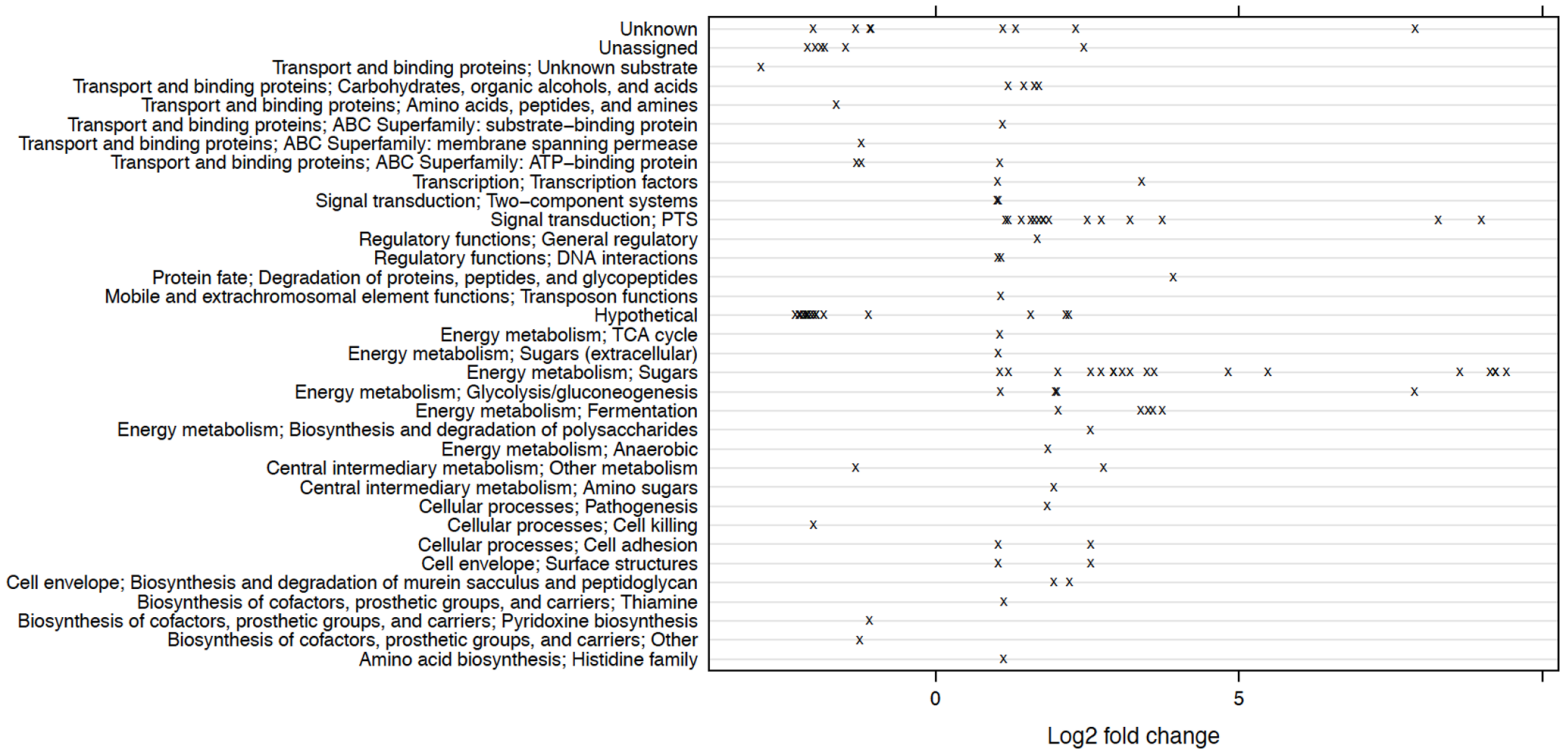
Distribution of functional classes of differentially expressed genes in UA159 growing in TV-glucose and TV-galactose. The *x*-axis represents the log_2_ values of the fold of change in expression (galactose/glucose).

Figure S6 shows proportions of functional categories that included differentially expressed genes between glucose-grown and galactose-grown UA159 cells. Of the four pairwise comparisons, this showed the largest number of genes, 101, as differentially expressed (Table 3). Again, energy metabolism had the most (30%) differentially expressed genes and as many as 16 PTS genes were affected, reflecting potential changes in uptake of lactose, galactose, glucose, fructose, mannose, cellobiose, sucrose and trehalose (Table S4). Clearly, glucose is a potent activator of CCR, negatively affecting the expression of various glycolytic components and PTS operons in the *S. mutans* genome. Notably in *S. mutans,* the effects of glucose on some of these PTS operons, including EII^Lac^ [47] EII^Cel^ [44] and EII^Fru^ (SMU.1956c∼1961c) [48] have been shown to be mediated primarily through the major glucose-PTS EII^Man^; and CcpA exerts its regulation indirectly by negatively regulating the expression of the genes for EII^Man^ [48]. Therefore, the transcriptomic alterations observed here are likely a result of these two tiers of regulation: one directly effected by CcpA and the other exerted through changes in the production of EII^man^. In addition, the profound changes in gene expression in the parental strain as a function of carbohydrate source was also attributable in part to the fact that galactose and its derivatives could serve as inducers of expression of the Leloir pathway and tagatose pathway genes that are responsible for assimilating galactose and lactose (Table S5) [47], [49]. These include the contiguous genes SMU.1486c to SMU.1496c (tagatose pathway) and genes SMU.885 to SMU.889 encoding the Leloir pathway. Interestingly, roughly 30% of all differentially expressed genes were considered hypothetical or of unknown function according to Oralgen database (http://oralgen.lanl.gov), and 74% of these were up-regulated in the presence of glucose (Figure 5). Among these were two clusters of genes that were up-regulated by glucose, SMU.150∼153 that encode the bacteriocin mutacin IV capable of killing closely related oral streptococci [50]; and the SMU.1898∼1914c gene cluster that includes two putative ABC-transporters, a putative bacteriocin-secretion protein (SMU.1905c), a bacteriocin-related protein (SMU.1906c) and a bacteriocin immunity protein (SMU.1913c).

Notably, genes that were expressed at a lower level when glucose was the growth carbohydrate rather than galactose included the *glg* operon (SMU.1535c∼1539c), the glucan-synthesizing enzymes *gtfBC* (SMU.1004∼1005) [51], [52], the pyruvate-formate lyase (SMU.402c), a glucan-binding protein *gbp*C (SMU.1396c) [53] and an NAD-dependent aldehyde dehydrogenase *gabD* (SMU.2127). Furthermore, a two-component system, *lytT* and *lytS* (SMU.576c∼577c) whose gene products are required for the expression of the genes for the bacterial holin:antiholin homologues LrgAB and for oxidative stress tolerance [54], [55], were down-regulated by the presence of glucose, although expression of *lrgAB* was not altered. Conversely, two additional genes encoding holin:antiholin-like proteins (SMU.1700c∼1701c, *cidB* and *cidA*, respectively) that were previously found inversely regulated in relation to *lrgAB* [55], were up-regulated in the presence of glucose.

Our previous Microarray analysis of the same set of samples showed similar patterns of change in gene expression, with a significant portion (28 out of 90) of the affected genes encoding hypothetical proteins [11]. However, in comparison with the predictions using Regprecise, the RNA-seq analysis identified 15 presumably CCR-sensitive operons, 8 of which were not detected in the Microarray study performed under identical conditions: SMU.112∼116 (fructose-1-phosphate kinase and a fructose-PTS EIIBC) [56], SMU.1088 (a putative thiamine biosynthesis lipoprotein), SMU.1843 (*scrB*), SMU.2127 (*gabD*), SMU.1596∼1600 (cellobiose operon), SMU.1877∼1879 (EII^Man^), SMU.980 (β-glucoside PTS EII) and the sucrose-PTS (*scrA*). Conversely, 8 operons from the list of Regprecise were identified by Microarray, but only two exclusively: SMU.574c∼575c (*lrgBA*) [55] and SMU.396 (a glycerol-uptake facilitator protein, *glpF*).

#### Comparison between glucose- and galactose-grown cells in the absence of CcpA

As mentioned above, the effect of carbohydrate source appears to have a greater impact on gene expression than does the loss of CcpA. Similar to the observations made with strain UA159, when the transcriptomes from strain TW1 grown in glucose or galactose were contrasted, transcripts for energy metabolism (45%) constituted the greatest proportion of differentially-expressed genes (Figure S7, S8, Tables S6, S7). In contrast to wild-type cells, however, only 7 PTS genes were identified that encode PTS components for lactose, fructose and mannitol (Table 4). Notably up-regulated by galactose in TW1 were the tagatose and Leloir pathways for galactose/lactose metabolism, the *glg* operon for glycogen metabolism, *gbpC, gtfB* and the *msm* pathway. A total of seven genes were found down-regulated in galactose-grown TW1 cells, with the majority being classified as hypothetical proteins.

Different from our previous Microarray study that saw as many as 515 genes differentially expressed by at least two-fold in TW1, with more than 50% of which having lower expression in galactose conditions, RNA-seq analysis performed at identical statistical thresholds showed only 42 genes in total affected in TW1 as a function of the growth carbohydrate. This divergence persisted when the analysis was performed under a lower threshold and a more relaxed *P* value (Table S8). A similar discrepancy was noted in the comparative analyses of the wild-type and *ccpA* mutant cells growing on glucose that were carried out using these two technologies, where markedly more genes were found to be down-regulated due to the loss of CcpA by Microarray than RNA-seq (see above). While some of these differential expressions have been confirmed by RealTime quantitative RT-PCR in our previous study ([11], and unpublished data), many more remain untested due to the large number of the affected genes, and the likelihood of many being indirectly regulated by CcpA. Multiple comparative studies by workers in other fields have suggested that as a likely replacement of Microarrays for high-throughput transcriptomic analysis, RNA-seq appears to be consistently more sensitive and repeatable [34]. Considering the many differences between these two methodologies that range from sample preparation, data generation and normalization, to statistical modeling that leads to identification of differential expression, these discrepancies noted in our study are likely the result of multiple factors. For example, whereas the Microarray study was carried out using total RNA, the RNA samples used in RNA-seq analysis were enriched for mRNA and other non-rRNA populations. Conversely, steps such as reverse transcription and adapter ligation are also known to affect the consistency of RNA-seq analysis. Nevertheless, since the difference between TW1 cells grown in glucose and galactose conditions is expected to be greatly reduced due to the loss of CcpA, these observations perhaps imply improved accuracy of RNA-seq technology over microarrays.

#### Comparison between the wild-type and *ccpA* mutant growing in TV-galactose

As reported previously, galactose is less effective than glucose at triggering CCR in *S. mutans* [48], so the finding that there were fewer differences between the transcriptomes of UA159 and TW1 in galactose-grown cells was expected. Nevertheless, energy metabolism remained the predominant category (41%) among all differentially expressed genes (Figures S9, S10). Similar to what was observed in glucose-grown cells, a few genes, including those for PDH components (SMU.1421c∼1425c) were up-regulated in TW1, and α-amylase (SMU.1590c) and β-D-fructosyltransferase *ftf* (SMU.2028c), were down-regulated (Table 5). In contrast, no major PTS genes were differentially expressed in the mutant during growth in galactose. Because both strains were grown in galactose, genes involved in galactose and lactose utilization were likely being expressed similarly and therefore no differences were noted. Consequently, no gene ontology terms were found significantly associated with differentially expressed genes. These results are largely consistent with our previous Microarray analysis, despite significantly more genes (57 genes) having been found to be down-regulated in the Microarray study [11].

## Concluding Remarks

Our current knowledge of the *S. mutans* transcriptome is limited to annotated or predicted genes. Whole transcriptome sequencing using high throughput sequencing technologies, deep RNA sequencing (RNA-seq), allowed us to better reveal the transcriptomic topography of *S. mutans*. Instead of aiming at pin-pointing transcript start or end sites, we focused on differential expression of genes in response to nutrient availability and deletion of *ccpA* from UA159. Although we did not use strand-specific RNA-seq, which could be important in gene-dense transcriptomes of species like bacteria or archaea, our application of a hidden Markov model for predicting transcripts to RNA-seq data in conjunction with known gene annotations allowed for inference of segments of putatively co-transcribed regions. In doing so, we attempted to ensure a high-quality transcriptome assembly. Although this map of transcripts did not precisely tell us where transcripts start and end, it does provide information as to which open reading frames could be co-transcribed under the conditions tested. The integration of this data with the newly developed genome browser described above is an asset for future analysis of the transcriptome of *S. mutans.*

In comparison with our previous Microarray study of differential gene expression using the same set of samples, we observed generally good agreement between these two methodologies, but were also able to identify genes by each method alone (especially RNA-seq) that appeared to match an *in silico* analysis published online at Regprecise website, and consistent with other independent studies [54], [55], [57], [58], [59], [60]. Further, additional genes and transcripts that are differentially regulated in *S. mutans* in response to carbohydrate source or loss of CcpA were identified. We also found discrepancies of annotated genes and their transcription, for example a gene containing two transcripts and non-coding regions producing significant numbers of transcripts. We hope that our work serves as a foundation for a comprehensive study of the *S. mutans* transcriptome and a more thorough evaluation of the role of non-coding sequences in gene regulation.

## Supporting Information

Figure S1 **Length distribution for expressed and unexpressed genes.** Darker gray bars represent frequency of expressed genes, and lighter gray ones that of unexpressed genes. The last bin sums from 3000 bp to 8500 bp.
(TIFF)

Figure S2 **Length distribution for expressed transcripts with annotated genes and unannotated genes.** Darker gray bars represent frequency of expressed transcripts with annotated genes, and lighter gray ones that of unannotated genes. The last bin sums from 10000 bp to 30000 bp.
(TIFF)

Figure S3 **Length distribution for 5' and 3' UTR.** Darker gray bars represent frequency of 5' UTR lengths, and lighter gray 3' UTR lengths. The last bin sums from 300 bp to 6500 bp.
(TIFF)

Figure S4 MFE structures drawing encoding positional entropy for differentially expressed regions.
(TIFF)

Figure S5 Distribution of functional classes of differentially expressed genes in UA159 and TW1 grown in glucose.
(TIFF)

Figure S6 Distribution of functional classes of differentially expressed genes in UA159 grown in glucose and galactose.
(TIFF)

Figure S7 Distribution of functional classes of differentially expressed genes in TW1 grown in glucose and galactose.
(TIFF)

Figure S8 Dot-chat distribution of functional classes of differentially expressed genes in TW1 grown in glucose and galactose.
(TIFF)

Figure S9 Distribution of functional classes of differentially expressed genes in UA159 and TW1 grown in galactose.
(TIFF)

Figure S10 Dot-chart distribution of functional classes of differentially expressed genes in UA159 and TW1 grown in galactose.
(TIFF)

Table S1 Neighboring genes of predicted small RNAs.
(PDF)

Table S2 Gene Ontology (GO) enrichments for differentially expressed genes in UA159 and TW1 grown in glucose.
(DOCX)

Table S3 KEGG enrichments for differentially expressed genes in UA159 and TW1 grown in glucose.
(DOCX)

Table S4 Gene Ontology (GO) enrichments for differentially expressed genes in UA159 grown in glucose and galactose.
(DOCX)

Table S5 KEGG enrichments for differentially expressed genes in UA159 grown in glucose and galactose.
(DOCX)

Table S6 Gene Ontology (GO) enrichments for differentially expressed genes in TW1 grown in glucose and galactose.
(DOCX)

Table S7 KEGG enrichments for differentially expressed genes in TW1 grown in glucose and galactose.
(DOCX)

Table S8 Differentially expressed genes in four pair-wise comparisons, analyzed under the threshold of > 1.5-fold of change in expression and adjusted *P* value of < 0.05.
(XLSX)
