# Supplementary figures and images for: Gene Regulation by CcpA and Catabolite Repression Explored by RNA-Seq in Streptococcus mutans
Source: PLoS One. 2013 Mar 28;8(3):e60465. doi: 10.1371/journal.pone.0060465 (PMC3610829; doi:10.1371/journal.pone.0060465)

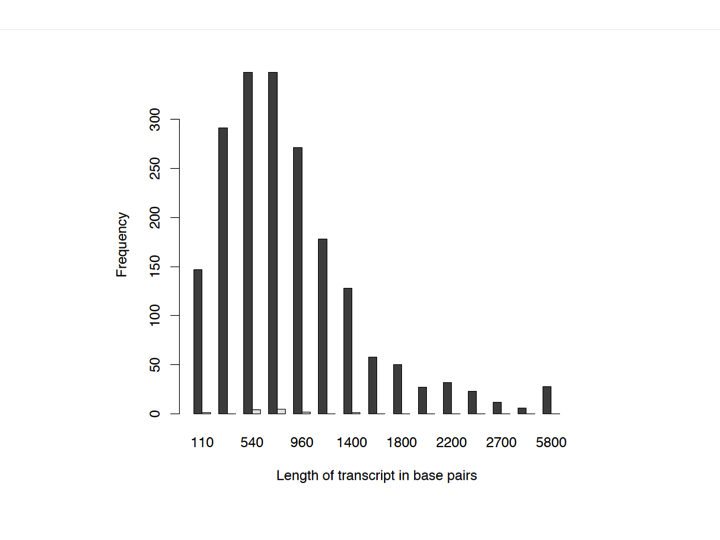

Supplement: Figure S1 — Length distribution for expressed and unexpressed genes. Darker gray bars represent frequency of expressed genes, and lighter gray ones that of unexpressed genes. The last bin sums from 3000 bp to 8500 bp. (TIFF) [file pone.0060465.s001.tiff]

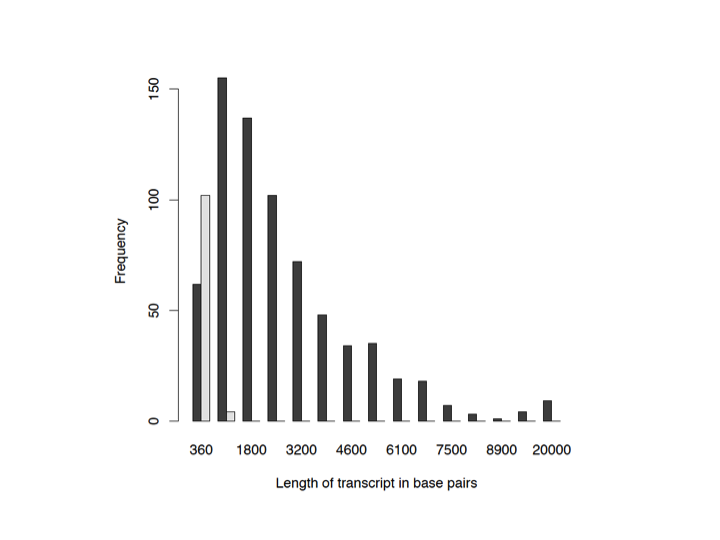

Supplement: Figure S2 — Length distribution for expressed transcripts with annotated genes and unannotated genes. Darker gray bars represent frequency of expressed transcripts with annotated genes, and lighter gray ones that of unannotated genes. The last bin sums from 10000 bp to 30000 bp. (TIFF) [file pone.0060465.s002.tiff]

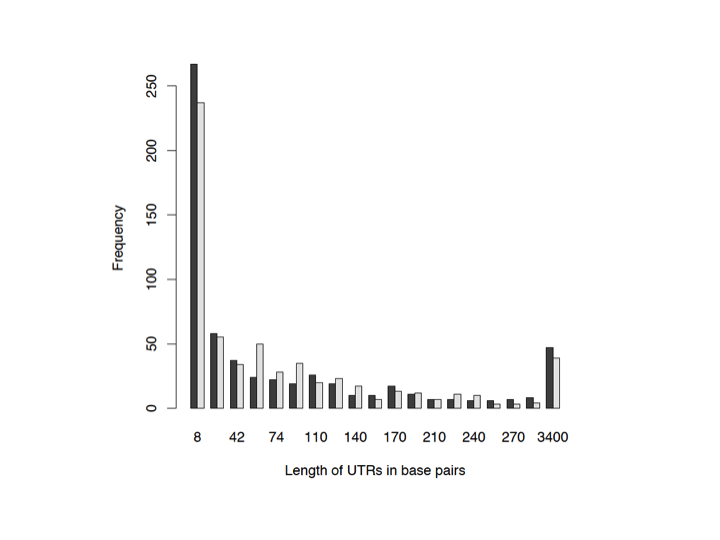

Supplement: Figure S3 — Length distribution for 5' and 3' UTR. Darker gray bars represent frequency of 5' UTR lengths, and lighter gray 3' UTR lengths. The last bin sums from 300 bp to 6500 bp. (TIFF) [file pone.0060465.s003.tiff]

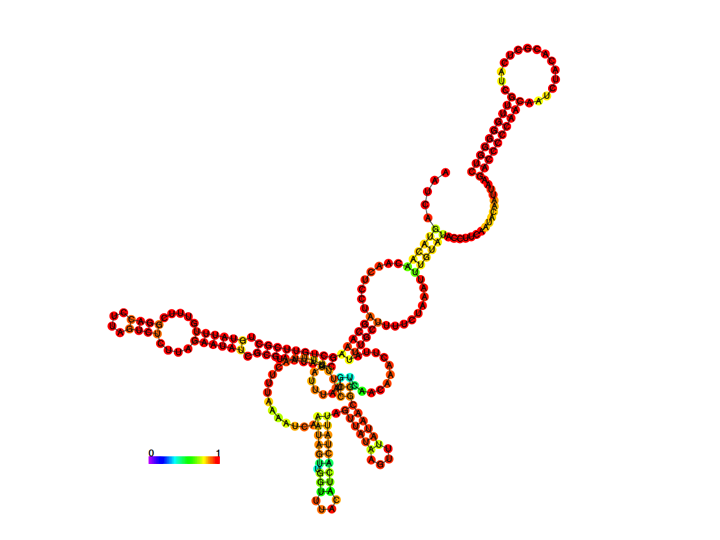

Supplement: Figure S4 — MFE structures drawing encoding positional entropy for differentially expressed regions. (TIFF) [file pone.0060465.s004.tiff]

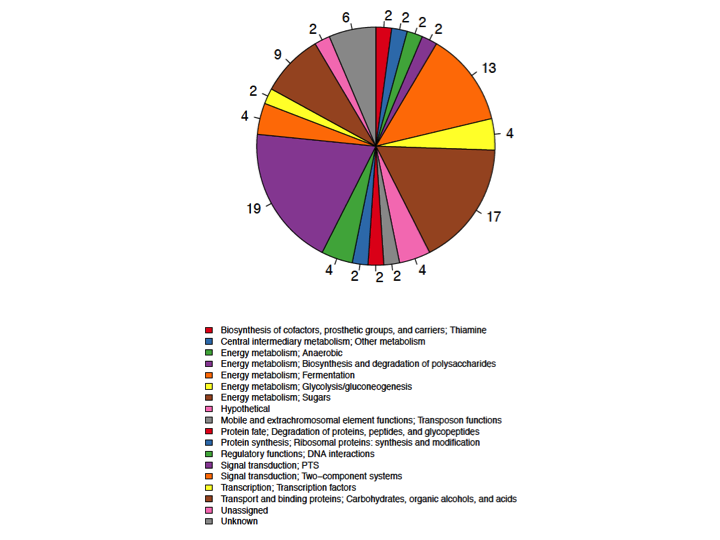

Supplement: Figure S5 — Distribution of functional classes of differentially expressed genes in UA159 and TW1 grown in glucose. (TIFF) [file pone.0060465.s005.tiff]

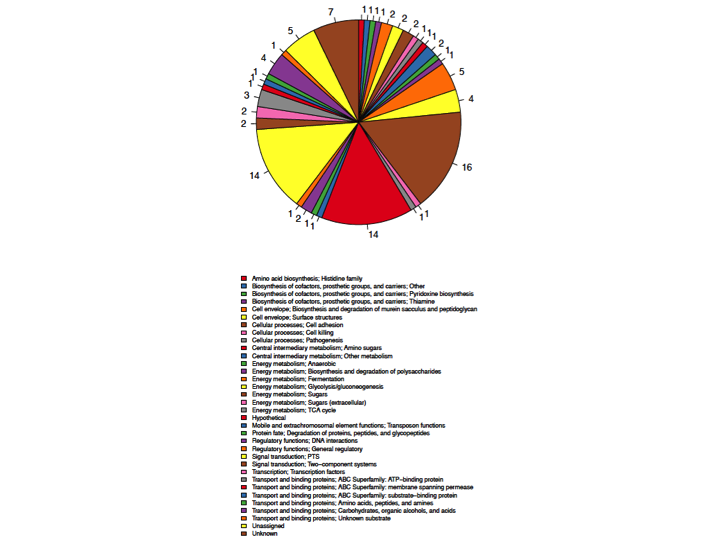

Supplement: Figure S6 — Distribution of functional classes of differentially expressed genes in UA159 grown in glucose and galactose. (TIFF) [file pone.0060465.s006.tiff]

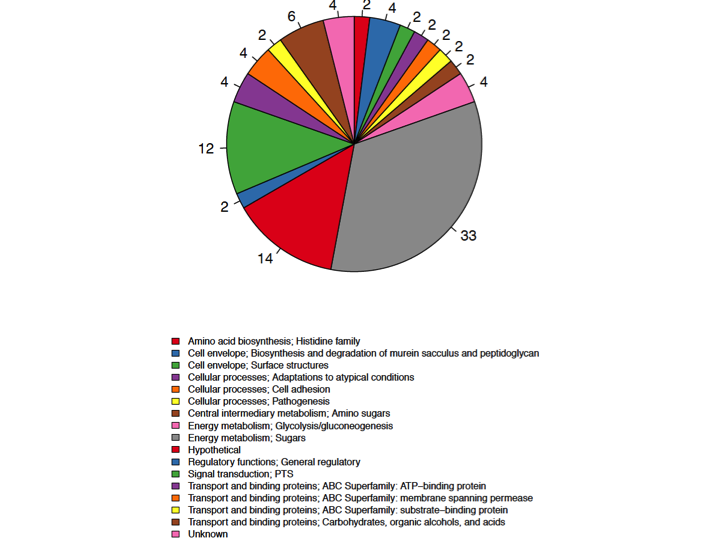

Supplement: Figure S7 — Distribution of functional classes of differentially expressed genes in TW1 grown in glucose and galactose. (TIFF) [file pone.0060465.s007.tiff]

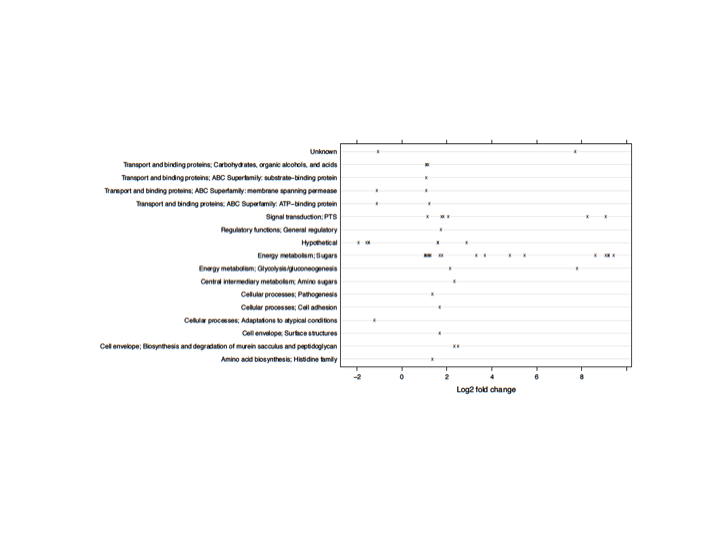

Supplement: Figure S8 — Dot-chat distribution of functional classes of differentially expressed genes in TW1 grown in glucose and galactose. (TIFF) [file pone.0060465.s008.tiff]

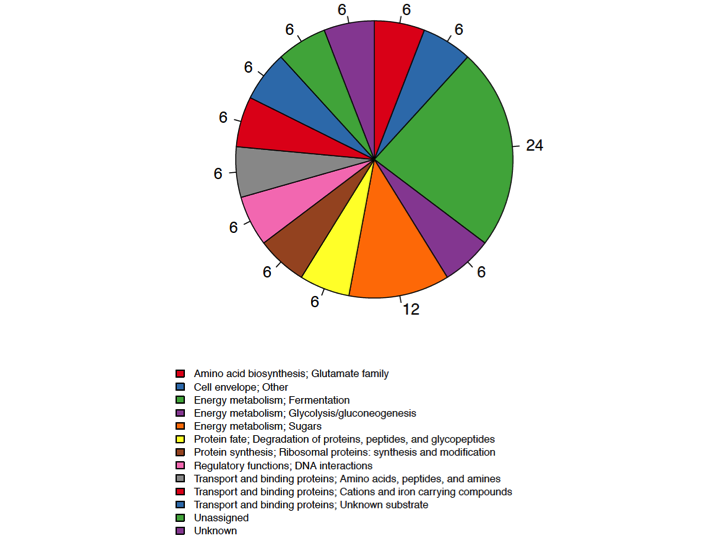

Supplement: Figure S9 — Distribution of functional classes of differentially expressed genes in UA159 and TW1 grown in galactose. (TIFF) [file pone.0060465.s009.tiff]

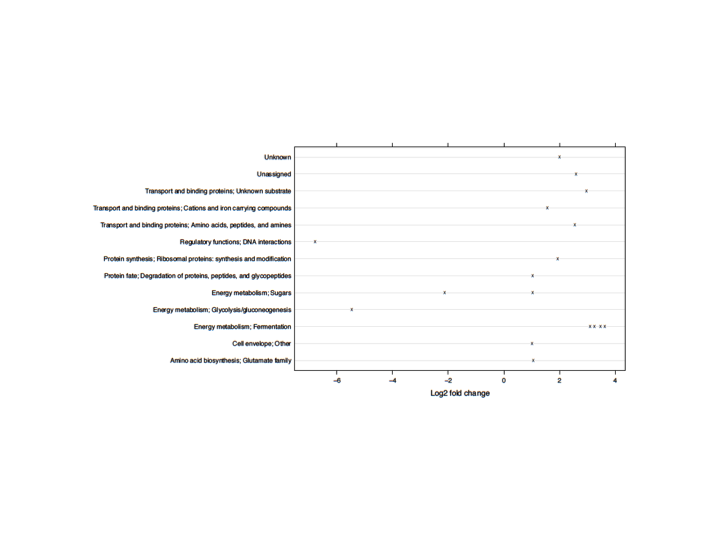

Supplement: Figure S10 — Dot-chart distribution of functional classes of differentially expressed genes in UA159 and TW1 grown in galactose. (TIFF) [file pone.0060465.s010.tiff]
