# Supplementary material for: Gene Regulation by CcpA and Catabolite Repression Explored by RNA-Seq in Streptococcus mutans
Source: PLoS One. 2013 Mar 28;8(3):e60465. doi: 10.1371/journal.pone.0060465 (PMC3610829; doi:10.1371/journal.pone.0060465)
Supplement: Table S1 — Neighboring genes of predicted small RNAs. (PDF) [file pone.0060465.s011.pdf]

Supplementary Table S1. Neighboring genes of predicted small RNAs

Description	Left gene	Distance	Predicted sRNA	Distance	Right gene	Description
			PredSmallRNA-1	29	SMU.01	chromosomal replication i ...
putative cell division pr ...	SMU.15	266	PredSmallRNA-2	83	SMU.16	putative amino acid permease
hypothetical protein	SMU.18	229	PredSmallRNA-3	6548	SMU.20	putative cell shape-deter ...
hypothetical protein	SMU.18	349	PredSmallRNA-4	6468	SMU.20	putative cell shape-deter ...
hypothetical protein	SMU.18	549	PredSmallRNA-5	6268	SMU.20	putative cell shape-deter ...
hypothetical protein	SMU.18	709	PredSmallRNA-6	6108	SMU.20	putative cell shape-deter ...
hypothetical protein	SMU.18	789	PredSmallRNA-7	6028	SMU.20	putative cell shape-deter ...
hypothetical protein	SMU.18	909	PredSmallRNA-8	5828	SMU.20	putative cell shape-deter ...
hypothetical protein	SMU.18	1229	PredSmallRNA-9	5588	SMU.20	putative cell shape-deter ...
hypothetical protein	SMU.18	1349	PredSmallRNA-10	5468	SMU.20	putative cell shape-deter ...
hypothetical protein	SMU.18	1429	PredSmallRNA-11	5388	SMU.20	putative cell shape-deter ...
hypothetical protein	SMU.18	1589	PredSmallRNA-12	5188	SMU.20	putative cell shape-deter ...
hypothetical protein	SMU.18	1789	PredSmallRNA-13	5046	SMU.20	putative cell shape-deter ...
hypothetical protein	SMU.18	2128	PredSmallRNA-14	4649	SMU.20	putative cell shape-deter ...
hypothetical protein	SMU.18	2288	PredSmallRNA-15	4530	SMU.20	putative cell shape-deter ...
hypothetical protein	SMU.18	2367	PredSmallRNA-16	4450	SMU.20	putative cell shape-deter ...
hypothetical protein	SMU.18	2447	PredSmallRNA-17	4330	SMU.20	putative cell shape-deter ...
hypothetical protein	SMU.18	3404	PredSmallRNA-18	3413	SMU.20	putative cell shape-deter ...
hypothetical protein	SMU.18	3524	PredSmallRNA-19	3293	SMU.20	putative cell shape-deter ...
hypothetical protein	SMU.18	3764	PredSmallRNA-20	3053	SMU.20	putative cell shape-deter ...
hypothetical protein	SMU.18	3884	PredSmallRNA-21	2933	SMU.20	putative cell shape-deter ...

hypothetical protein	SMU.18	4164	PredSmallRNA-22	2653	SMU.20	putative cell shape-deter ...
hypothetical protein	SMU.18	4284	PredSmallRNA-23	2533	SMU.20	putative cell shape-deter ...
hypothetical protein	SMU.18	4364	PredSmallRNA-24	2413	SMU.20	putative cell shape-deter ...
hypothetical protein	SMU.18	4524	PredSmallRNA-25	2253	SMU.20	putative cell shape-deter ...
hypothetical protein	SMU.18	4724	PredSmallRNA-26	2053	SMU.20	putative cell shape-deter ...
hypothetical protein	SMU.18	4879	PredSmallRNA-27	1938	SMU.20	putative cell shape-deter ...
hypothetical protein	SMU.39	83	PredSmallRNA-28	44	SMU.40	hypothetical protein
DNA alkylation repair enzyme	SMU.60	210	PredSmallRNA-29	60	SMU.61	putative transcriptional ...
putative N-acetyl-muramidase	SMU.76	105	PredSmallRNA-30	281	SMU.78	fructan hydrolase; exo-be ...
CTP synthetase	SMU.97	234	PredSmallRNA-31	508	SMU.99	fructose-bisphosphate aldolase
CTP synthetase	SMU.97	749	PredSmallRNA-32	132	SMU.99	fructose-bisphosphate aldolase
putative putative transposase	SMU.106c	578	PredSmallRNA-33	391	SMU.107	hypothetical protein
putative putative transposase	SMU.106c	903	PredSmallRNA-34	33	SMU.107	hypothetical protein
hypothetical protein	SMU.153	406	PredSmallRNA-35	97	SMU.154	30S ribosomal protein S15
30S ribosomal protein S15	SMU.154	68	PredSmallRNA-36	410	SMU.155	polynucleotide phosphoryl ...
polynucleotide phosphoryl ...	SMU.155	-164	PredSmallRNA-37	-64	SMU.156	hypothetical protein
putative transcriptional ...	SMU.168	134	PredSmallRNA-38	138	SMU.169	50S ribosomal protein L13
hypothetical protein	SMU.189	669	PredSmallRNA-39	5302	SMU.191c	putative integrase
hypothetical protein	SMU.189	1109	PredSmallRNA-40	5182	SMU.191c	putative integrase
hypothetical protein	SMU.189	1229	PredSmallRNA-41	5062	SMU.191c	putative integrase
hypothetical protein	SMU.189	1349	PredSmallRNA-42	4862	SMU.191c	putative integrase
hypothetical protein	SMU.189	1549	PredSmallRNA-43	4662	SMU.191c	putative integrase
hypothetical protein	SMU.189	1983	PredSmallRNA-44	3798	SMU.191c	putative integrase

hypothetical protein	SMU.189	2647	PredSmallRNA-45	3644	SMU.191c	putative integrase
hypothetical protein	SMU.189	2767	PredSmallRNA-46	3524	SMU.191c	putative integrase
hypothetical protein	SMU.189	3524	PredSmallRNA-47	2767	SMU.191c	putative integrase
hypothetical protein	SMU.189	3884	PredSmallRNA-48	2407	SMU.191c	putative integrase
hypothetical protein	SMU.189	4524	PredSmallRNA-49	1727	SMU.191c	putative integrase
hypothetical protein	SMU.189	4724	PredSmallRNA-50	1567	SMU.191c	putative integrase
hypothetical protein	SMU.189	4844	PredSmallRNA-51	1440	SMU.191c	putative integrase
hypothetical protein	SMU.189	5426	PredSmallRNA-52	755	SMU.191c	putative integrase
hypothetical protein	SMU.217c	139	PredSmallRNA-53	58	SMU.218	putative transcriptional ...
hypothetical protein	SMU.219	179	PredSmallRNA-54	49	SMU.220c	hypothetical protein
hypothetical protein	SMU.220c	111	PredSmallRNA-55	50	SMU.221c	putative integrase
putative integrase	SMU.221c	30	PredSmallRNA-56	318	SMU.222c	integrase fragment
putative amino acid ABC t ...	SMU.242c	236	PredSmallRNA-57	266	SMU.243	hypothetical protein
hypothetical protein	SMU.281	583	PredSmallRNA-58	142	SMU.283	hypothetical protein
hypothetical protein	SMU.285	173	PredSmallRNA-59	35	SMU.286	putative ABC transporter, ...
putative transcriptional ...	SMU.289	182	PredSmallRNA-60	186	SMU.290	putative L-ascorbate 6-ph ...
putative tetrahydrodipico ...	SMU.317	468	PredSmallRNA-61	174	SMU.318	putative hippurate hydrolase
putative hippurate hydrolase	SMU.318	38	PredSmallRNA-62	182	SMU.320	putative 5-formyltetrahyd ...
DNA repair protein RadA	SMU.327	-755	PredSmallRNA-63	611	SMU.328	putative carbonic anhydrase
hypothetical protein	SMU.329	73	PredSmallRNA-64	99	SMU.330	glutamyl-tRNA synthetase
argininosuccinate synthase	SMU.334	61	PredSmallRNA-65	55	SMU.335	argininosuccinate lyase
hypothetical protein	SMU.350	219	PredSmallRNA-66	245	SMU.351	ribosome-associated GTPase
phosphoglycerate kinase	SMU.361	107	PredSmallRNA-67	200	SMU.362	hypothetical protein

phosphoglycerate kinase	SMU.361	275	PredSmallRNA-68	46	SMU.362	hypothetical protein
hypothetical protein	SMU.369c	222	PredSmallRNA-69	67	SMU.370	putative ABC transporter, ...
pyruvate formate-lyase	SMU.402	96	PredSmallRNA-70	74	SMU.403	DNA polymerase IV
hypothetical protein	SMU.423	49	PredSmallRNA-71	136	SMU.424	negative transcriptional ...
hypothetical protein	SMU.442	86	PredSmallRNA-72	50	SMU.444	hypothetical protein
hypothetical protein	SMU.471	210	PredSmallRNA-73	25	SMU.472	N6-adenine-specific DNA m ...
putative type II restrict ...	SMU.506	226	PredSmallRNA-74	47	SMU.507	DeoR family transcription ...
hypothetical protein	SMU.512c	67	PredSmallRNA-75	189	SMU.513	hypothetical protein
hypothetical protein	SMU.513	89	PredSmallRNA-76	51	SMU.514	putative transcriptional ...
putative transcriptional ...	SMU.526c	40	PredSmallRNA-77	162	SMU.527	hypothetical protein
hypothetical protein	SMU.530c	446	PredSmallRNA-78	44	SMU.531	putative chorismate mutase
tryptophan synthase subun ...	SMU.538	55	PredSmallRNA-79	122	SMU.539c	signal peptidase type IV
putative cell division pr ...	SMU.557	66	PredSmallRNA-80	219	SMU.558	isoleucyl-tRNA synthetase
isoleucyl-tRNA synthetase	SMU.558	93	PredSmallRNA-81	526	SMU.560c	hypothetical protein
isoleucyl-tRNA synthetase	SMU.558	401	PredSmallRNA-82	145	SMU.560c	hypothetical protein
hypothetical protein	SMU.600c	264	PredSmallRNA-83	42	SMU.602	putative sodium-dependent ...
ATP-dependent RNA helicase	SMU.611	110	PredSmallRNA-84	165	SMU.613	hypothetical protein
hypothetical protein	SMU.614	433	PredSmallRNA-85	191	SMU.616	hypothetical protein
putative manganese-type s ...	SMU.629	58	PredSmallRNA-86	100	SMU.630	hypothetical protein
putative esterase	SMU.643	93	PredSmallRNA-87	52	SMU.644	putative competence prote ...
foldase protein PrsA	SMU.648	44	PredSmallRNA-88	53	SMU.649	hypothetical protein
ribonucleotide-diphosphat ...	SMU.667	428	PredSmallRNA-89	64	SMU.668c	ribonucleotide-diphosphat ...
putative glutaredoxin	SMU.669c	73	PredSmallRNA-90	44	SMU.670	aconitate hydratase

hypothetical protein	SMU.706c	37	PredSmallRNA-91	34	SMU.707c	putative endolysin
elongation factor Tu	SMU.714	3	PredSmallRNA-92	225	SMU.715	triosephosphate isomerase
hypothetical protein	SMU.722	84	PredSmallRNA-93	7	SMU.723	putative calcium-transpor ...
putative glycerophosphory ...	SMU.724	156	PredSmallRNA-94	61	SMU.725c	hypothetical protein
hypothetical protein	SMU.739c	90	PredSmallRNA-95	55	SMU.741	hypothetical protein
putative manganese transporter	SMU.770c	70	PredSmallRNA-96	155	SMU.771c	hypothetical protein
hypothetical protein	SMU.771c	57	PredSmallRNA-97	110	SMU.772	putative glucan-binding p ...
putative RNA methyltransferase	SMU.788	349	PredSmallRNA-98	118	SMU.789	hypothetical protein
GTPase ObgE	SMU.801	43	PredSmallRNA-99	175	SMU.802	hypothetical protein
hypothetical protein	SMU.807	72	PredSmallRNA-100	76	SMU.809	excinuclease ABC subunit B
hypothetical protein	SMU.812	59	PredSmallRNA-101	74	SMU.813	transcription regulator
hypothetical protein	SMU.851	391	PredSmallRNA-102	46	SMU.852	putative transcriptional ...
hypothetical protein	SMU.855	77	PredSmallRNA-103	43	SMU.856	bifunctional pyrimidine r ...
putative transposase, IS1 ...	SMU.875c	235	PredSmallRNA-104	114	SMU.876	putative MSM operon regul ...
hypothetical protein	SMU.941c	98	PredSmallRNA-105	108	SMU.942	putative hydroxymethylglu ...
putative Clp-like ATP-dep ...	SMU.956	132	PredSmallRNA-106	66	SMU.957	50S ribosomal protein L10
putative dehydrogenase	SMU.962	128	PredSmallRNA-107	81	SMU.963c	putative deacetylase
tRNA (uracil-5-)-methyltr ...	SMU.1003	129	PredSmallRNA-108	47	SMU.1004	glucosyltransferase-I
glucosyltransferase-Si	SMU.1005	123	PredSmallRNA-109	43	SMU.1006	putative ABC transporter, ...
putative hydrolase or acy ...	SMU.1028	165	PredSmallRNA-110	130	SMU.1029	hypothetical protein
putative transposon integ ...	SMU.1032	158	PredSmallRNA-111	190	SMU.1034c	site-specific tyrosine re ...
cell wall protein, WapE	SMU.1091	74	PredSmallRNA-112	204	SMU.1093	putative ABC transporter, ...
putative permease	SMU.1100c	55	PredSmallRNA-113	113	SMU.1102	6-phospho-beta-glucosidase

phosphoglycerate mutase-1 ...	SMU.1106c	247	PredSmallRNA-114	32	SMU.1107c	hypothetical protein
hypothetical protein	SMU.1153c	149	PredSmallRNA-115	145	SMU.1154c	hypothetical protein
ATP-dependent DNA helicase	SMU.1174	221	PredSmallRNA-116	76	SMU.1175	putative sodium/amino aci ...
PTS system, mannitol-spec ...	SMU.1185	120	PredSmallRNA-117	192	SMU.1187	glucosamine–fructose-6-p ...
hypothetical protein	SMU.1189c	64	PredSmallRNA-118	65	SMU.1190	pyruvate kinase
hypothetical protein	SMU.1197	76	PredSmallRNA-119	280	SMU.1200	30S ribosomal protein S1
dihydroorotate dehydrogen ...	SMU.1224	39	PredSmallRNA-120	113	SMU.1225	putative transcriptional ...
restriction endonuclease	SMU.1259	135	PredSmallRNA-121	56	SMU.1260c	hypothetical protein
1-(5-phosphoribosyl)-5-[(...	SMU.1265	161	PredSmallRNA-122	65	SMU.1266	imidazole glycerol phosph ...
histidinol-phosphate amin ...	SMU.1273	18	PredSmallRNA-123	917	SMU.1276c	septation ring formation ...
histidinol-phosphate amin ...	SMU.1273	410	PredSmallRNA-124	352	SMU.1276c	septation ring formation ...
histidinol-phosphate amin ...	SMU.1273	849	PredSmallRNA-125	84	SMU.1276c	septation ring formation ...
putative transcriptional ...	SMU.1282	114	PredSmallRNA-126	80	SMU.1284c	hypothetical protein
putative dipeptidase	SMU.1303c	158	PredSmallRNA-127	89	SMU.1304c	hypothetical protein
hypothetical protein	SMU.1317c	317	PredSmallRNA-128	395	SMU.1319c	hypothetical protein
hypothetical protein	SMU.1317c	700	PredSmallRNA-129	52	SMU.1319c	hypothetical protein
4Fe-4S ferredoxin	SMU.1327c	39	PredSmallRNA-130	75	SMU.1329c	putative transposase
putative transposase	SMU.1332c	89	PredSmallRNA-131	636	SMU.1334	putative phosphopantethei ...
putative transposase	SMU.1332c	208	PredSmallRNA-132	205	SMU.1334	putative phosphopantethei ...
hypothetical protein	SMU.1349	89	PredSmallRNA-133	44	SMU.1351	putative putative transposase
putative transposase	SMU.1353	93	PredSmallRNA-134	199	SMU.1354c	putative putative transposase
putative transposase	SMU.1363c	115	PredSmallRNA-135	169	SMU.1365c	permease
putative transposase	SMU.1363c	269	PredSmallRNA-136	88	SMU.1365c	permease

hypothetical protein	SMU.1367c	117	PredSmallRNA-137	115	SMU.1368	hypothetical protein
putative transposase, IS1 ...	SMU.1370c	235	PredSmallRNA-138	293	SMU.1372c	hypothetical protein
hypothetical protein	SMU.1374	45	PredSmallRNA-139	152	SMU.1375c	hypothetical protein
2-isopropylmalate synthase	SMU.1384	13	PredSmallRNA-140	58	SMU.1386	uridine kinase
hypothetical protein	SMU.1389	98	PredSmallRNA-141	80	SMU.1390	hypothetical protein
hypothetical protein	SMU.1397c	93	PredSmallRNA-142	162	SMU.1398	putative transcriptional ...
hypothetical protein	SMU.1405c	50	PredSmallRNA-143	241	SMU.1406c	hypothetical protein
putative dihydrolipoamide ...	SMU.1424	70	PredSmallRNA-144	58	SMU.1425	putative Clp proteinase, ...
putative Zn-dependent protease	SMU.1438c	364	PredSmallRNA-145	39	SMU.1442c	hypothetical protein
putative tributyrin esterase	SMU.1443c	30	PredSmallRNA-146	407	SMU.1444c	hypothetical protein
adenine phosphoribosyltra ...	SMU.1467	99	PredSmallRNA-147	361	SMU.1470c	hypothetical protein
galactose-6-phosphate iso ...	SMU.1496	128	PredSmallRNA-148	57	SMU.1498	lactose repressor
lactose repressor	SMU.1498	130	PredSmallRNA-149	54	SMU.1499	putative exonuclease RexA
phenylalanyl-tRNA synthet ...	SMU.1512	46	PredSmallRNA-150	164	SMU.1513	putative chromosome segre ...
hypothetical protein	SMU.1577c	55	PredSmallRNA-151	72	SMU.1578	biotin–protein ligase
hypothetical protein	SMU.1587c	41	PredSmallRNA-152	35	SMU.1588c	putative hexosyltransferase
catabolite control protei ...	SMU.1591	49	PredSmallRNA-153	56	SMU.1592	putative dipeptidase PepQ
SsrA-binding protein	SMU.1606	-253	PredSmallRNA-154	5	SMU.1607	putative exoribonuclease ...
putative exoribonuclease ...	SMU.1607	31	PredSmallRNA-155	71	SMU.1609c	preprotein translocase su ...
putative metalloprotease	SMU.1619c	111	PredSmallRNA-156	73	SMU.1620	putative phosphate starva ...
methionyl-tRNA synthetase	SMU.1639	401	PredSmallRNA-157	60	SMU.1641c	hypothetical protein
D-alanine–poly(phosphori ...	SMU.1691	71	PredSmallRNA-158	500	SMU.1692	pyruvate-formate lyase ac ...
D-alanine–poly(phosphori ...	SMU.1691	371	PredSmallRNA-159	212	SMU.1692	pyruvate-formate lyase ac ...

putative rRNA methylase	SMU.1707c	51	PredSmallRNA-160	67	SMU.1708	potassium transporter per ...
hypothetical protein	SMU.1719c	66	PredSmallRNA-161	141	SMU.1721c	putative diaminopimelate ...
OxaA-like protein precursor	SMU.1727	75	PredSmallRNA-162	109	SMU.1728	transcription elongation ...
putative transcriptional ...	SMU.1745c	23	PredSmallRNA-163	84	SMU.1746c	enoyl-CoA hydratase
aspartate kinase	SMU.1748	287	PredSmallRNA-164	6665	SMU.1750c	hypothetical protein
aspartate kinase	SMU.1748	1677	PredSmallRNA-165	5388	SMU.1750c	hypothetical protein
aspartate kinase	SMU.1748	2117	PredSmallRNA-166	4948	SMU.1750c	hypothetical protein
aspartate kinase	SMU.1748	2917	PredSmallRNA-167	4188	SMU.1750c	hypothetical protein
aspartate kinase	SMU.1748	3197	PredSmallRNA-168	3911	SMU.1750c	hypothetical protein
aspartate kinase	SMU.1748	3274	PredSmallRNA-169	3831	SMU.1750c	hypothetical protein
aspartate kinase	SMU.1748	3674	PredSmallRNA-170	3391	SMU.1750c	hypothetical protein
aspartate kinase	SMU.1748	3875	PredSmallRNA-171	2992	SMU.1750c	hypothetical protein
aspartate kinase	SMU.1748	4233	PredSmallRNA-172	2679	SMU.1750c	hypothetical protein
aspartate kinase	SMU.1748	4625	PredSmallRNA-173	2493	SMU.1750c	hypothetical protein
aspartate kinase	SMU.1748	5132	PredSmallRNA-174	1893	SMU.1750c	hypothetical protein
aspartate kinase	SMU.1748	5452	PredSmallRNA-175	1653	SMU.1750c	hypothetical protein
aspartate kinase	SMU.1748	5852	PredSmallRNA-176	1253	SMU.1750c	hypothetical protein
aspartate kinase	SMU.1748	6052	PredSmallRNA-177	973	SMU.1750c	hypothetical protein
aspartate kinase	SMU.1748	6332	PredSmallRNA-178	784	SMU.1750c	hypothetical protein
aspartate kinase	SMU.1748	6666	PredSmallRNA-179	429	SMU.1750c	hypothetical protein
hypothetical protein	SMU.1752c	30	PredSmallRNA-180	53	SMU.1753c	hypothetical protein
hypothetical protein	SMU.1764c	69	PredSmallRNA-181	132	SMU.1765c	hypothetical protein
hypothetical protein	SMU.1768c	158	PredSmallRNA-182	261	SMU.1770	valyl-tRNA synthetase

hypothetical protein	SMU.1773c	66	PredSmallRNA-183	150	SMU.1774c	hypothetical protein
hypothetical protein	SMU.1774c	36	PredSmallRNA-184	385	SMU.1775c	hypothetical protein
hypothetical protein	SMU.1774c	187	PredSmallRNA-185	229	SMU.1775c	hypothetical protein
hypothetical protein	SMU.1782	123	PredSmallRNA-186	5902	SMU.1783	prolyl-tRNA synthetase
hypothetical protein	SMU.1782	1102	PredSmallRNA-187	4946	SMU.1783	prolyl-tRNA synthetase
hypothetical protein	SMU.1782	1662	PredSmallRNA-188	4466	SMU.1783	prolyl-tRNA synthetase
hypothetical protein	SMU.1782	1902	PredSmallRNA-189	4266	SMU.1783	prolyl-tRNA synthetase
hypothetical protein	SMU.1782	2302	PredSmallRNA-190	3866	SMU.1783	prolyl-tRNA synthetase
hypothetical protein	SMU.1782	2422	PredSmallRNA-191	3706	SMU.1783	prolyl-tRNA synthetase
hypothetical protein	SMU.1782	3420	PredSmallRNA-192	2510	SMU.1783	prolyl-tRNA synthetase
hypothetical protein	SMU.1782	3778	PredSmallRNA-193	2212	SMU.1783	prolyl-tRNA synthetase
hypothetical protein	SMU.1782	4170	PredSmallRNA-194	2011	SMU.1783	prolyl-tRNA synthetase
hypothetical protein	SMU.1782	4397	PredSmallRNA-195	1771	SMU.1783	prolyl-tRNA synthetase
hypothetical protein	SMU.1782	4677	PredSmallRNA-196	1451	SMU.1783	prolyl-tRNA synthetase
hypothetical protein	SMU.1782	5157	PredSmallRNA-197	1011	SMU.1783	prolyl-tRNA synthetase
hypothetical protein	SMU.1782	5397	PredSmallRNA-198	771	SMU.1783	prolyl-tRNA synthetase
hypothetical protein	SMU.1782	5637	PredSmallRNA-199	491	SMU.1783	prolyl-tRNA synthetase
hypothetical protein	SMU.1782	5771	PredSmallRNA-200	186	SMU.1783	prolyl-tRNA synthetase
aspartyl-tRNA synthetase	SMU.1822	24	PredSmallRNA-201	144	SMU.1823	putative pyrazinamidase/n ...
excinuclease ABC subunit A	SMU.1851	274	PredSmallRNA-202	49	SMU.1852	putative magnesium/cobalt ...
hypothetical protein	SMU.1861c	93	PredSmallRNA-203	169	SMU.1862	hypothetical protein
hypothetical protein	SMU.1862	128	PredSmallRNA-204	674	SMU.1865	putative A/G-specific DNA ...
hypothetical protein	SMU.1862	857	PredSmallRNA-205	94	SMU.1865	putative A/G-specific DNA ...

putative ABC transporter, ...	SMU.1881c	125	PredSmallRNA-206	203	SMU.1882c	hypothetical protein
hypothetical protein	SMU.1882c	58	PredSmallRNA-207	59	SMU.1883	hypothetical protein
hypothetical protein	SMU.1884c	148	PredSmallRNA-208	148	SMU.1886	seryl-tRNA synthetase
hypothetical protein	SMU.1900	60	PredSmallRNA-209	90	SMU.1902c	hypothetical protein
hypothetical protein	SMU.1907	78	PredSmallRNA-210	46	SMU.1908c	hypothetical protein
hypothetical protein	SMU.1910c	252	PredSmallRNA-211	238	SMU.1912c	hypothetical protein
putative response regulat ...	SMU.1917	66	PredSmallRNA-212	242	SMU.1918	putative membrane-associa ...
response regulator GcrR f ...	SMU.1924	181	PredSmallRNA-213	134	SMU.1925c	hypothetical protein
hypothetical protein	SMU.1951c	35	PredSmallRNA-214	308	SMU.1954	chaperonin GroEL
hypothetical protein	SMU.1951c	395	PredSmallRNA-215	123	SMU.1954	chaperonin GroEL
putative PTS system, suga ...	SMU.1961c	143	PredSmallRNA-216	58	SMU.1963c	putative sugar-binding pe ...
DNA-directed RNA polymera ...	SMU.1990	82	PredSmallRNA-217	35	SMU.1991	putative membrane carboxy ...
putative membrane carboxy ...	SMU.1991	38	PredSmallRNA-218	52	SMU.1992	tyrosyl-tRNA synthetase
putative transcriptional ...	SMU.1995c	67	PredSmallRNA-219	349	SMU.1996	4-diphosphocytidyl-2-C-me ...
putative transcriptional ...	SMU.1995c	549	PredSmallRNA-220	32	SMU.1996	4-diphosphocytidyl-2-C-me ...
putative ComX1, transcrip ...	SMU.1997	578	PredSmallRNA-221	5223	SMU.1999c	hypothetical protein
putative ComX1, transcrip ...	SMU.1997	1154	PredSmallRNA-222	4774	SMU.1999c	hypothetical protein
putative ComX1, transcrip ...	SMU.1997	1554	PredSmallRNA-223	4294	SMU.1999c	hypothetical protein
putative ComX1, transcrip ...	SMU.1997	1834	PredSmallRNA-224	4094	SMU.1999c	hypothetical protein
putative ComX1, transcrip ...	SMU.1997	2194	PredSmallRNA-225	3734	SMU.1999c	hypothetical protein
putative ComX1, transcrip ...	SMU.1997	2354	PredSmallRNA-226	3534	SMU.1999c	hypothetical protein
putative ComX1, transcrip ...	SMU.1997	2514	PredSmallRNA-227	3297	SMU.1999c	hypothetical protein
putative ComX1, transcrip ...	SMU.1997	3351	PredSmallRNA-228	2537	SMU.1999c	hypothetical protein

putative ComX1, transcrip ...	SMU.1997	3551	PredSmallRNA-229	2338	SMU.1999c	hypothetical protein
putative ComX1, transcrip ...	SMU.1997	3710	PredSmallRNA-230	2218	SMU.1999c	hypothetical protein
putative ComX1, transcrip ...	SMU.1997	4609	PredSmallRNA-231	1279	SMU.1999c	hypothetical protein
putative ComX1, transcrip ...	SMU.1997	5289	PredSmallRNA-232	599	SMU.1999c	hypothetical protein
putative ComX1, transcrip ...	SMU.1997	5489	PredSmallRNA-233	319	SMU.1999c	hypothetical protein
hypothetical protein	SMU.2033c	134	PredSmallRNA-234	430	SMU.2035	bacteriocin immunity protein
recombination factor prot ...	SMU.2056	129	PredSmallRNA-235	51	SMU.2057c	putative cadmium-transpor ...
putative stress response ...	SMU.2067	406	PredSmallRNA-236	46	SMU.2069	zinc transporter ZupT
putative short-chain dehy ...	SMU.2115	261	PredSmallRNA-237	144	SMU.2116	putative osmoprotectant a ...
tRNA-specific 2-thiouridy ...	SMU.2143c	71	PredSmallRNA-238	119	SMU.2146c	hypothetical protein
transmembrane protein	SMU.2160	133	PredSmallRNA-239	396	SMU.2161c	hypothetical protein
transmembrane protein	SMU.2160	435	PredSmallRNA-240	206	SMU.2161c	hypothetical protein
rRNA large subunit methyl ...	SMU.2162c	33	PredSmallRNA-241	89	SMU.2164	serine protease HtrA
serine protease HtrA	SMU.2164	126	PredSmallRNA-242	200	SMU.2165	putative SpoJ
serine protease HtrA	SMU.2164	374	PredSmallRNA-243	36	SMU.2165	putative SpoJ
