# Supplementary material for: Gene Regulation by CcpA and Catabolite Repression Explored by RNA-Seq in Streptococcus mutans
Source: PLoS One. 2013 Mar 28;8(3):e60465. doi: 10.1371/journal.pone.0060465 (PMC3610829; doi:10.1371/journal.pone.0060465)
Supplement: Table S2 — Gene Ontology (GO) enrichments for differentially expressed genes in UA159 and TW1 grown in glucose. (DOCX) [file pone.0060465.s012.docx]

| ***q*^a^** | **Description** |
| --- | --- |
| 2.619073e-05 | phosphoenolpyruvate-dependent sugar phosphotransferase system |
| 0.0009471108 | protein-N(PI)-phosphohistidine-sugar phosphotransferase activity |

**Table S2. Gene Ontology (GO) enrichments for differentially expressed genes in UA159 and TW1 grown in glucose.**

^a^ We used a multiple-testing adjusted p-value of 10^-3^ to determine differentially expressed genes. For each category having at least ten genes a variation of Fisher's exact test was performed, and another multiple-testing adjusted p-value, or q-value was obtained. The listed categories were chosen at the cutoff value of 10^-3^.
