# Supplementary material for: Gene Regulation by CcpA and Catabolite Repression Explored by RNA-Seq in Streptococcus mutans
Source: PLoS One. 2013 Mar 28;8(3):e60465. doi: 10.1371/journal.pone.0060465 (PMC3610829; doi:10.1371/journal.pone.0060465)
Supplement: Table S4 — Gene Ontology (GO) enrichments for differentially expressed genes in UA159 grown in glucose and galactose. (DOCX) [file pone.0060465.s014.docx]

| ***q*^a^** | **Description** |
| --- | --- |
| 5.892253e-07 | Carbohydrate metabolic process |
| 5.892253e-07 | Carbohydrate transport |
| 2.524915e-06 | Phosphoenolpyruvate-dependent sugar phosphotransferase system |
| 2.567088e-05 | Lactose metabolic process |
| 5.842943e-05 | Sugar:hydrogen symporter activity |
| 6.256511e-05 | Protein-N(PI)-phosphohistidine-sugar phosphotransferase activity |

**Table S4. Gene Ontology (GO) enrichments for differentially expressed genes in UA159 grown in glucose and galactose.**

^a^ We used a multiple-testing adjusted p-value of 10^-3^ to determine differentially expressed genes. For each category having at least ten genes a variation of Fisher's exact test was performed, and another multiple-testing adjusted p-value, or q-value was obtained. The listed categories were chosen at the cutoff value of 10^-3^.
