# Supplementary material for: Gene Regulation by CcpA and Catabolite Repression Explored by RNA-Seq in Streptococcus mutans
Source: PLoS One. 2013 Mar 28;8(3):e60465. doi: 10.1371/journal.pone.0060465 (PMC3610829; doi:10.1371/journal.pone.0060465)
Supplement: Table S5 — KEGG enrichments for differentially expressed genes in UA159 grown in glucose and galactose. (DOCX) [file pone.0060465.s015.docx]

| ***q*^a^** | **Description** |
| --- | --- |
| 7.25873e-11 | Galactose metabolism [PATH:smu00052] |
| 2.025638e-08 | Phosphotransferase system (PTS) [PATH:smu02060] |
| 6.856151e-06 | Starch and sucrose metabolism [PATH:smu00500] |
| 0.0006327025 | Fructose and mannose metabolism [PATH:smu00051] |
| 0.0008674382 | Amino sugar and nucleotide sugar metabolism [PATH:smu00520] |

**Table S5. KEGG enrichments for differentially expressed genes in UA159 grown in glucose and galactose.**

^a^ We used a multiple-testing adjusted p-value of 10^-3^ to determine differentially expressed genes. For each category having at least ten genes a variation of Fisher's exact test was performed, and another multiple-testing adjusted p-value, or q-value was obtained. The listed categories were chosen at the cutoff value of 10^-3^.
