# Supplementary material for: Gene Regulation by CcpA and Catabolite Repression Explored by RNA-Seq in Streptococcus mutans
Source: PLoS One. 2013 Mar 28;8(3):e60465. doi: 10.1371/journal.pone.0060465 (PMC3610829; doi:10.1371/journal.pone.0060465)
Supplement: Table S7 — KEGG enrichments for differentially expressed genes in TW1 grown in glucose and galactose. (DOCX) [file pone.0060465.s017.docx]

| ***q*^a^** | **Description** |
| --- | --- |
| 0 | Galactose metabolism [PATH:smu00052] |
| 0.00065503 | Starch and sucrose metabolism [PATH:smu00500] |

**Table S7. KEGG enrichments for differentially expressed genes in TW1 grown in glucose and galactose.**

^a^ We used a multiple-testing adjusted p-value of 10^-3^ to determine differentially expressed genes. For each category having at least ten genes a variation of Fisher's exact test was performed, and another multiple-testing adjusted p-value, or q-value was obtained. The listed categories were chosen at the cutoff value of 10^-3^.
